# Stabilization of Weakly Unstable Fixed Points as a Common Dynamical Mechanism of High-Frequency Electrical Stimulation

**DOI:** 10.1038/s41598-020-62839-6

**Published:** 2020-04-03

**Authors:** Dan Wilson

**Affiliations:** Department of Electrical Engineering and Computer Science, University of Tennessee, Knoxville, TN 37996 USA

**Keywords:** Computational models, Dynamical systems, Electrical and electronic engineering, Applied mathematics

## Abstract

While high-frequency electrical stimulation often used to treat various biological diseases, it is generally difficult to understand its dynamical mechanisms of action. In this work, high-frequency electrical stimulation is considered in the context of neurological and cardiological systems. Despite inherent differences between these systems, results from both theory and computational modeling suggest identical dynamical mechanisms responsible for desirable qualitative changes in behavior in response to high-frequency stimuli. Specifically, desynchronization observed in a population of periodically firing neurons and reversible conduction block that occurs in cardiomyocytes both result from bifurcations engendered by stimulation that modifies the stability of unstable fixed points. Using a reduced order phase-amplitude modeling framework, this phenomenon is described in detail from a theoretical perspective. Results are consistent with and provide additional insight for previously published experimental observations. Also, it is found that sinusoidal input is energy-optimal for modifying the stability of weakly unstable fixed points using periodic stimulation.

## Introduction

High-frequency electrical stimulation (HFES) has been widely investigated as a possible therapeutic treatment for various diseases. Among specific applications, deep brain stimulation (DBS) is a well-established technique for alleviating the movement symptoms of Parkinson’s disease^1–3^, whereby high-frequency electrical pulses are injected into an appropriate region of the brain; DBS has also shown promise as a potential treatment for other neurological disorders such as Depression^4^, Tourette syndrome^5^, and Alzheimer’s Disease^6^. Additionally, it has long been known that high-frequency alternating current can be used to reversibly block peripheral nerve conduction^7–9^, leading to the suggestion that such a strategy could be used to temporarily block muscle spasms and pain. Sinusoidal, high-frequency electric fields have also been investigated in the context of inducing reversible cardiac conduction block^10–12^ with potential applications to cardiac defibrillation. HFES is particularly attractive in a clinical setting because it can be applied in a mostly open-loop manner: a clinician can apply the stimulation, check whether symptoms have abated, and adjust stimulation parameters until satisfactory control is achieved.

From a physiological perspective, the mechanisms underlying the observed response to HFES are usually reasonably well understood. For example, the application of DBS induces some combination of activation, inhibition, or desynchronization^13–16^ in neural populations that influence larger brain circuits. By contrast, the underlying dynamical mechanisms responsible for the effects caused by HFES are not well-studied. In this work, theoretical analysis and supporting computational simulations suggest that the influence of HFES in various applications can be characterized by a common dynamical mechanism. Specifically, in different high-dimensional computational models describing cardiological and neurological electrophysiology, high-frequency stimulation is found to induce qualitative changes in the stability of fixed points with linearizations that have near-zero principle eigenvalues. Consequently, with an appropriate combination of stimulus frequency, period, and shape, the sign of the eigenvalues can be changed to produce qualitative changes in the model behavior and produce desired effects. In contrast to other theoretical results that are valid in the limit of high frequency forcing^12,17–20^ the proposed mechanism is valid in the limit of small magnitude forcing allowing lower frequency inputs to be considered. Additionally, the theoretical analysis to follow is performed using a reduced coordinate framework that allows particularly high-dimensional models to be considered.

Investigation of two distinct applications provide support for this argument. In the first application, HFES applied to a numerical model describing a population of neurons (e.g., as implemented during DBS) is considered. In conjunction with complicated spatial patterns of activation and inhibition of neural cell bodies, axons, and dendrites induced by DBS^13,14^, evidence suggests that high-frequency DBS disrupts pathological synchronization that contributes to the motor symptoms of Parkinson’s disease^15,16,21^, which has motivated the search for more efficient desynchronizing stimulation protocols^22,23^. In the second application, HFES as applied to a numerical model of the myocardium and potential applications to defibrillation are considered. Experimental studies have found that the application of high-frequency alternating current to individual cardiomyocytes can indefinitely prolong repolarization resulting in reversible conduction block^10,11^ that could be used in alternative defibrillation strategies. Numerical modeling and analysis^12^ in a minimal cardiomyocyte model identified various ionic currents which participate in this prolongation of repolarization, however the fundamental mechanisms for this observed behavior in more realistic models has not been explored.

In the results to follow, the ability of periodic, charge-balanced electrical stimulation to stabilize a weakly unstable fixed point, thereby engendering changes in the qualitative dynamical behavior is investigated. From a theoretical perspective, using phase-amplitude reduction strategies and the notion of isostable coordinates as a starting point^24,25^, it is found that sinusoidal forcing is energy-optimal for modifying the stability of a fixed point of a general set of ordinary differential equations. Computational simulations and subsequent theoretical analysis provides evidence that desynchronization in a large population of neural oscillators and repolarization block in individual cardiomyocites in response to high-frequency stimulation may be governed by the stabilization of underlying unstable fixed points. This work suggests a novel dynamical mechanism that is consistent across a wide variety of applications under which HFES operates; this framework could be used to develop new treatments that incorporate HFES.

## Results

### Main theoretical results

Consider a forced nonlinear ordinary differential equation

1 $$\dot{x}=F(x)+g(t),$$

where $x\in {{\mathbb{R}}}^{n}$, *F* gives the nominal dynamics, *g*(*t*) = [*u*(*t*) 0 … 0]^*T*^ is an ${\mathcal{O}}(\epsilon )$*T*-periodic, exogenous input and 0≤*ϵ* ≪ 0. Suppose that when *g*(*t*) = 0, *x*_0_ is a fixed point of (1) with eigenvalues *λ*_1_, …, *λ*_*n*_ ordered so that ${\rm{Re}}({\lambda }_{i})\le {\rm{Re}}({\lambda }_{i+1})$. Given the large dimension of the models considered in this work, model reduction strategies will be used to asses stability changes resulting from periodic stimulation. Specifically, a phase-amplitude reduced coordinate system will be used^24,25^ in order to assess the influence of inputs. To do so, let *u*(*t*) = *u*_0_(*t*) + Δ*u*(*t*) where *u*_0_(*t*) represents a nominal *T*-periodic input and Δ*u*(*t*) is some deviation from this nominal input. A *T*-periodic periodic orbit resulting from *u*_0_(*t*) will emerge and the behavior of this periodic orbit can be analyzed in terms of its phase and isostable coordinates

2 $$\begin{array}{lll}\dot{\theta } & = & \omega +\left(Z(\theta )+\mathop{\sum }\limits_{k=1}^{n}{\psi }_{k}{B}^{k}(\theta )\right)\Delta u(t),\\ {\dot{\psi }}_{j} & = & {\kappa }_{j}{\psi }_{j}+\left({I}_{j}(\theta )+\mathop{\sum }\limits_{k=1}^{n}{\psi }_{k}{C}_{j}^{k}(\theta )\right)\Delta u(t),\\ j & = & 1,\ldots ,n-1.\end{array}$$

Here, *θ* is the phase of oscillation which gives a sense of the location on the periodic orbit, *ψ*_*j*_ is the *j*^th^ isostable coordinate which gives a sense of the distance from the periodic orbit, *ω* = 2*π*/*T* is the natural frequency, *κ*_*j*_ is a Floquet exponent, *Z*(*θ*) and *I*_*j*_(*θ*) are the phase and isostable response curves, respectively, which characterize the first order accurate dynamics of the phase and isostable coordinates in response to external perturbations, and *B*^*k*^(*θ*) and ${C}_{j}^{k}(\theta )$ provide second-order accurate corrections. Note here that the periodic orbit itself is induced by the nominal periodic forcing *u*_0_(*t*) and as such, *u*_0_(*t*) is assumed not to be zero for all time. More details about the transformation of (1) to (2) are presented in the Methods section. The stability of the periodic orbit can be determined by the Floquet exponents^26^ which govern the growth of transient solutions near the orbit; if all Floquet exponents are negative, the periodic orbit is stable, otherwise it is unstable. As shown in the Methods section, the set of Floquet exponents *κ*_*j*_ for *j* = 1, …, *n* depend on the stimulation *u*(*t*) according to

3 $${\kappa }_{j}={\lambda }_{j}+\mathop{\sum }\limits_{k=1}^{\infty }{\alpha }_{j}\left(T/k\right)\left({a}_{k}^{2}+{b}_{k}^{2}\right),$$

where *a*_*k*_ and *b*_*k*_ are the *k*^th^ terms of the Fourier series expansion of *u*(*t*) (i.e., ${a}_{k}=\frac{2}{T}{\int }_{0}^{T}u(t)\cos (2\pi kt/T)dt$ and ${b}_{k}=\frac{2}{T}{\int }_{0}^{T}u(t)\sin (2\pi kt/T)dt$, and *α*_*j*_(*T*/*k*) is a function based on underlying system dynamics (see Eq. (54) and its associated derivation for an explanation of the terms comprising each *α*_*j*_). Compared with strategies used in^27,28^, Eq. (3) directly identifies the change in Floquet exponents resulting from the stimulation *u*(*t*). Additionally, in contrast to other strategies that have been employed to understand the behavior of dynamical systems in response to periodic stimulation^12,17–20^, Eq. (3) is valid even for low frequencies (but requires that *u*(*t*) is small).

While Eq. (3) is valid for each eigenvalue, it is often the case that a single eigenvalue (or pair of complex eigenvalues) are unstable. In this case, if one can design a stimulus to modify the stability of that eigenvalue, the resulting periodic orbit can be stabilized.

#### Sinusoidal Stimulation is Energy Optimal to Stabilize an Unstable Fixed Point

As a consequence of (3) in many cases, purely sinusoidal stimulation is energy optimal for stabilizing an unstable fixed point. To see this, suppose that a given unstable fixed point ${x}_{{\rm{u}}}\in {{\mathbb{R}}}^{n}$ of (1) has only one unstable eigenvalue (or one unstable complex conjugate pair of eigenvalues) with an associated *α*_*j*_(*T*) that has negative real components for some values of *T*. Suppose also that all other eigenvalues are negative and large enough in magnitude so their stability cannot be changed with periodic forcing. If it is desired to stabilize *x*_u_ with an energy-optimal, periodic stimulus where energy consumption is defined as ${\mathcal{M}}={\int }_{0}^{T}{u}^{2}(t)dt$. Any zero-mean, continuous, piecewise smooth, and periodic function *u*(*t*) can be represented in terms of a Fourier basis $u(t)={\sum }_{k=1}^{\infty }[\sqrt{{a}_{k}^{2}+{b}_{k}^{2}}\sin (2\pi kt/T+{\zeta }_{k})]$, where *a*_*k*_ and *b*_*k*_ are Fourier coefficients and *ζ*_*k*_ is an offset determined by the the relative sizes of *a*_*k*_ and *b*_*k*_. Using Parseval’s theorem^29^, the average energy consumed by the stimulus can be written as

4 $$\frac{1}{T}{\int }_{0}^{T}{u}^{2}(t)dt=\frac{1}{2}{\sum }_{k=1}^{\infty }\left({a}_{k}^{2}+{b}_{k}^{2}\right).$$

This information can be used to show that purely sinusoidal input is energy optimal for stabilizing the unstable eigenvalue in the limit of small forcing. To show this, let ${E}_{0}\in {{\mathbb{R}}}^{+}$ be a fixed average energy consumption and *T*_*m*_ be an upper bound on the stimulus period. Stability of the resulting forced periodic orbit is determined by the sign of the real component of *κ*_1_. Starting with (3)

5 $$\begin{array}{lll}{\rm{Real}}({\kappa }_{1}) & \ge  & {\rm{Real}}({\lambda }_{1})+{\min }_{y\le {T}_{m}}({\rm{Real}}({\alpha }_{1}(y)))\mathop{\sum }\limits_{k=1}^{\infty }({a}_{k}^{2}+{b}_{k}^{2})\\  & = & {\lambda }_{1}+2{E}_{0}\ {\min }_{y\le {T}_{m}}({\rm{Real}}({\alpha }_{1}(y))).\end{array}$$

Equation (5) sets a lower bound on the real component of the resulting Floquet multiplier *κ*_1_. This lower bound can be achieved by taking $T={{\rm{argmin}}}_{y\le {T}_{m}}({\rm{Real}}({\alpha }_{1}(y)))$, letting ${a}_{1}^{2}+{b}_{1}^{2}={E}_{0}$, and letting both *a*_*k*_ and *b*_*k*_ equal zero for *k*≥2, i.e., by applying purely sinusoidal stimulus at the frequency with the period that minimizes *α*_1_. Other stimuli with identical average energy consumption *E*_0_ will result in larger real components of the Floquet exponent. For instance, such a stimulus with period *T* taken such that ${\alpha }_{1}(T)\ne {\min }_{y\le {T}_{m}}({\rm{Real}}({\alpha }_{1}(y)))$ with ${a}_{1}^{2}+{b}_{1}^{2} > 0$, the resulting real component of *κ*_1_ will be strictly greater than ${\lambda }_{1}+2{E}_{0}{\min }_{y\le {T}_{m}}({\rm{Real}}({\alpha }_{1}(y)))$. Additionally, if  $T={{\rm{argmin}}}_{y\le {T}_{m}}({\rm{Real}}({\alpha }_{1}(y)))$ but ${\sum }_{k=2}^{\infty }({a}_{k}^{2}+{b}_{k}^{2}) > 0$ the resulting real component of *κ*_1_ will also be strictly greater than ${\lambda }_{1}+2{E}_{0}{\min }_{y\le {T}_{m}}({\rm{Real}}({\alpha }_{1}(y)))$.

In order to stabilize a system with a single unstable fixed point, one requires Real(*κ*_1_) < 0, and in the limit that the magnitude of the stimulus is asymptotically small, the energy optimal strategy to drive the Floquet exponent toward more negative values is to stimulate with purely sinusoidal input with a period that minimizes Real(*α*_1_(*T*)). Other cost functions could be considered that may reflect other considerations such as limiting transfer of Faradaic charge^30^ or setting absolute limits on the magnitude of the applied stimulus^31^. These considerations would result in different optimal control stimuli that are not purely sinusoidal in most cases.

### Desynchronization in a large population of coupled neurons with high-frequency stimulation

It is well-known that patients with Parkinson’s disease display abnormally increased power in the beta range of local field potential recordings^32,33^ (at approximately 13–35 Hz). Furthermore, it has been shown that abatement of motor symptoms from therapeutically effective DBS is strongly correlated with a decrease in power in the beta band of local field potential recordings from patients with Parkinson’s disease. Taken together, these findings have led to the hypothesis that pathological neural synchronization contributes to the symptoms of Parkinson’s disease^3,21^, and that therapeutic DBS disrupts this undesirable brain rhythm.

External forcing in oscillatory dynamical systems has been widely studied. Entrainment to external periodic stimulation is commonly observed in populations of externally forced oscillators. Using phase reduced equations it is possible to identify optimal stimuli for entrainment to an external input^34,35^. The aggregate response of populations of oscillators to periodic external inputs has also been investigated in theoretical^36^ and detailed computational studies^37^. Further, synchronization among individual oscillators in response to common, aperiodic forcing has also been previously investigated in detail^38–40^. Previous authors have investigated a theoretical basis for desynchronization occurring for uncoupled populations of neurons^41,42^ in response to periodic inputs. Related methods were developed to engender chaotic desynchronization in a population of identical oscillators^43,44^. Others have investigated dynamical behavior of spiking neurons in the limit of high-frequency forcing^19,20^ illustrating that high frequency stimulation can suppress periodic firing in neurons. The results presented below suggest that at certain frequencies, external periodic input can be used to desynchronize populations of oscillators that are synchronized by coupling. Furthermore, in numerical simulations presented here it is observed that the underlying mechanism responsible for the desynchronization is the stabilization of an underlying unstable fixed point. While desynchronization in response to a common input has been seen in other applications, the dynamical mechanism proposed here has not been explored in detail.

To illustrate these results consider a large population of coupled conductance-based neurons from^45^

6 $$\begin{array}{lll}C{\dot{V}}_{j} & = & {f}_{V}({V}_{j},{h}_{j},{r}_{j})+{I}_{b}+{I}_{{\rm{stim}}}(t)+\sqrt{2D}{\eta }_{j}+\frac{1}{N}\mathop{\sum }\limits_{i=1}^{N}{\sigma }_{c}({V}_{i}-{V}_{j}),\\ {\dot{h}}_{j} & = & {f}_{h}({V}_{j},{h}_{j}),\\ {\dot{r}}_{j} & = & {f}_{r}({V}_{j},{r}_{j}),\quad j=1,\ldots ,N.\end{array}$$

Here, *V*_*j*_ is the transmembrane voltage of neuron *j*, *h*_*j*_ and *r*_*j*_ are associated gating variables, functions and parameters comprising *f*_*V*_, *f*_*h*_, and *f*_*r*_ are taken to be identical to those from^45^, *I*_*b*_ = 5 *μ*A/cm^2^ is a baseline current,  *C* = 1*μ*F/cm^2^ is the neural membrane capacitance, $\sqrt{2D}{\eta }_{j}$ is an independent and identically distributed zero-mean white noise process with intensity *D* = 1, ${I}_{{\rm{stim}}}(t)$ is an external current stimulus common to each neuron, and neurons are coupled electrotonically^46^. Note that all-to-all electrotonic coupling is used here to make the subsequent mathematical analysis more straightforward; in principle other types of neural coupling (e.g., synaptic^47^) could be used. In the absence of coupling and noise, for the parameters used here the limiting neural behavior is tonic firing with a period of *T* = 8.39 ms.

To simplify the analysis, it will be assumed that each individual neuron can be represented as a noisy limit cycle oscillator that obeys

7 $$\begin{array}{lll}{\dot{\theta }}_{j} & = & \omega +Z({\theta }_{j})\left({I}_{{\rm{stim}}}(t)+\sqrt{2D}{\eta }_{j}+\frac{1}{N}{\sum }_{i=1}^{N}{\sigma }_{c}(V({\theta }_{i})-V({\theta }_{j}))\right),\\ j & = & 1,\ldots ,N.\end{array}$$

**Figure 1:**
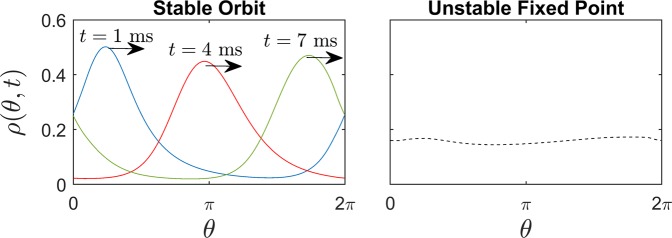
The left panel shows a stable periodic orbit of (8) which represents its unperturbed infinite time behavior. For this solution, the probability distribution travels from left to right in a highly synchronized manner. The right panel shows an unstable fixed point for the same model with a nearly uniform (desynchronized) distribution.

In (7), each oscillator from (6) has been phase reduced^48,49^, where *θ*_*j*_ ∈ [0, 2*π*) is the 2*π*-periodic phase of oscillator *j*, *ω* = 2*π*/*T* is the natural frequency where *T* is the unperturbed firing rate, and *Z*(*θ*) is the neuron’s phase response curve which characterizes how the phase changes in response to external stimuli. A fundamental assumption of (7) is that the Floquet multipliers of each neuron’s limit cycle are small enough in magnitude and resulting perturbations transverse to the limit cycle decay rapidly so that transmembrane voltage can be represented as a function of the phase. As part of (7), it is assumed that *D* is small enough for higher order noise terms to be neglected^50^. Furthermore, assuming that the population of neurons is large one can view (7) in terms of a probability density *ρ*(*θ*, *t*) evolving according to the Fokker-Planck equation^51^:

8 $$\begin{array}{lll}\frac{\partial \rho (\theta ,t)}{\partial t} & =& -\frac{\partial }{\partial \theta }[(\omega +Z(\theta)({I}_{{\rm{stim}}}(t)+{\sigma }_{c}(\bar{V}-V(\theta ))))\rho(\theta ,t)]+\frac{{\partial }^{2}}{\partial {\theta}^{2}}[D{Z}^{2}(\theta )\rho (\theta ,t)],\\  & = & {\underbrace{-\omega{\rho }_{\theta }+\frac{{\partial }^{2}}{\partial {\theta}^{2}}[D{Z}^{2}(\theta )\rho ]-\frac{\partial }{\partial \theta}[Z(\theta ){\sigma }_{c}(\bar{V}-V(\theta ))\rho]}_{{\rm{unperturbed}}\,{\rm{dynamics}}}}{\underbrace{-[Z(\theta){\rho }_{\theta }+{Z}^{{\prime} }(\theta )\rho]{I}_{{\rm{stim}}}(t)}_{{\rm{external}}\,{\rm{forcing}}}}\end{array}$$

where $\bar{V}={\int }_{0}^{2\pi }V(\theta )\rho (\theta )d\theta$ is the population averaged voltage. The boundary conditions of the PDE (8) are periodic. In the above equations, a distinction is made between the unperturbed dynamics and external forcing. This PDE model will be analyzed using a finite difference approximation with 400 total gridpoints. Upon discretization the resulting ordinary differential equations take the form:

9 $$\dot{x}=F(x)-{I}_{{\rm{stim}}}(t)({\rm{diag}}({Z}_{\theta })A+{\rm{diag}}({Z}_{\theta }^{{\prime} }))x,$$

where *x* is a vector of the discretized probability distribution, *F*(*x*) represents the resulting dynamics that are solely a function of the state (i.e., the unperturbed dynamics), *Z*_*θ*_ and ${Z}_{\theta }^{{\prime} }$ are discretized vectors of *Z*(*θ*) (the phase response curve of a single neuron to voltage perturbations) and its first derivative, respectively, and *A* is a matrix which implements the finite difference approximation of the derivative with respect to *θ*. For this particular model, taking *D* = 1 and *σ*_*c*_ = 0.08, when ${I}_{{\rm{stim}}}=0$ the stable steady state behavior is a periodic orbit as shown in the left panel of Fig. 1 with period 8.17 ms. The right panel of Fig. 1 shows an unstable fixed point of this model with a nearly uniform (desynchronized) distribution. The first few eigenvalues associated with this unstable fixed point are [*λ*_1,2_ *λ*_3,4_] = [0.007 ± 0.774*i* − 0.0711 ± 1.566*i*]. This unstable fixed point of (9) has only a single eigenvalue pair with positive real component; all other eigenvalues are stable.

Upon discretization of (8) the resulting ordinary differential equations (9) have a slightly different form than (1). Nevertheless, using an appropriate coordinate transformation, one can show that (3) still holds for this model. To see this, assuming that ${I}_{{\rm{stim}}}$ is an order *ϵ* perturbation, near an unperturbed fixed point *x*_0_ of (9) (i.e. with *F*(*x*_0_) = 0) the leading order *ϵ* dynamics are

10 $$\dot{x}=F(x)-{I}_{{\rm{stim}}}(t){r}_{0}+{\mathcal{O}}({\epsilon }^{2}),$$

where ${r}_{0}=({\rm{diag}}({Z}_{\theta })A+{\rm{diag}}({Z}_{\theta }^{{\prime} })){x}_{0}$ is a constant vector. By choosing any nonsingular matrix *V* such that *Vr*_0_ = [10 … 0]^*T*^, one can apply the coordinate transformation *y* = *Vx* with (10) to yield

11 $$\dot{y}=VF({V}^{-1}\ y)-{I}_{{\rm{stim}}}(t)V{r}_{o}+{\mathcal{O}}({\epsilon }^{2}).$$

Equation (11) is in the same form as (1) and therefore the result (3) also holds for the finite difference approximation of (8).

**Figure 2:**
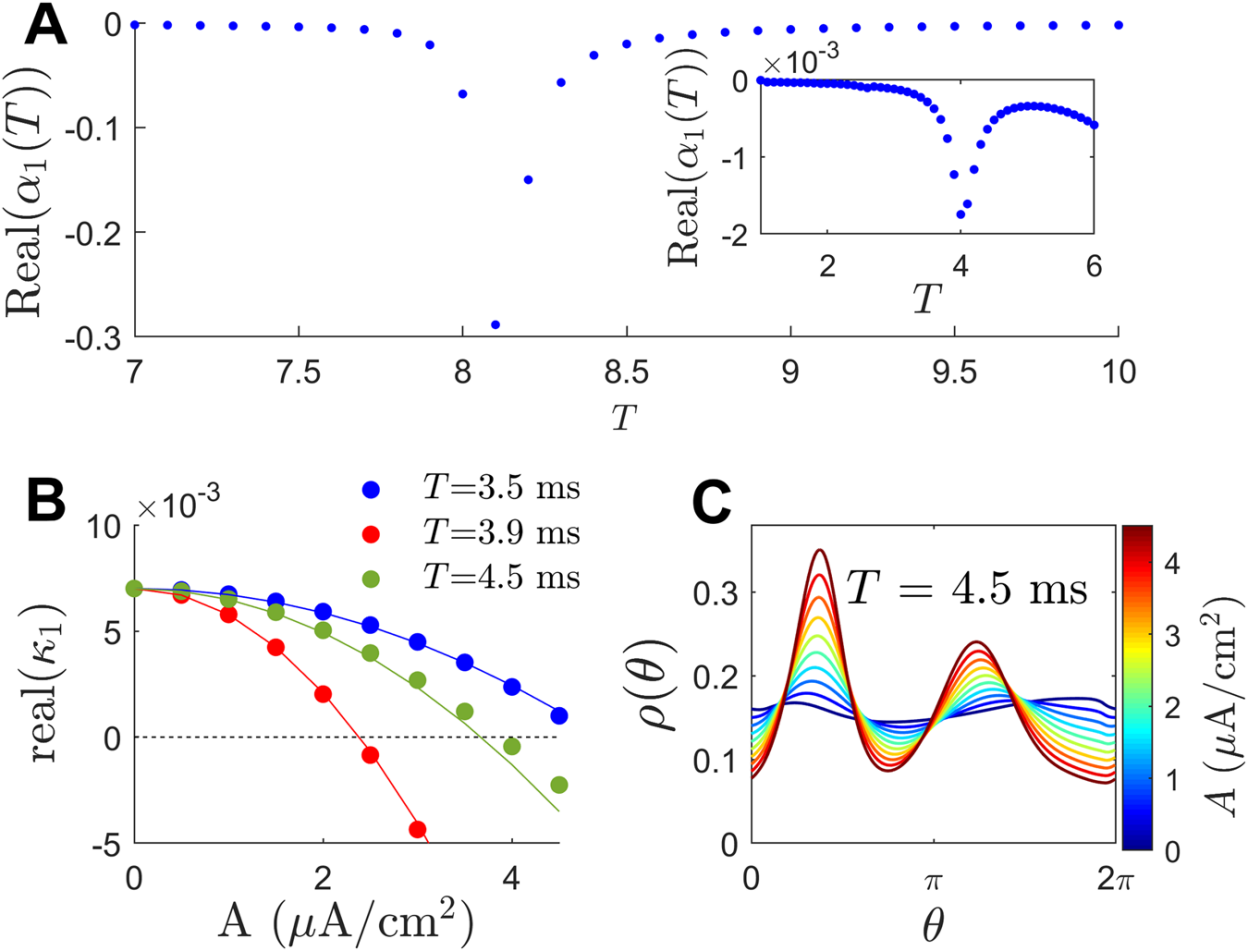
Behavior of unstable solutions of the Fokker-Planck equation (8) with sinusoidal forcing. Panel A shows a plot of Real(*α*_1_(*T*)) which characterizes the ability of sinusoidal stimulation to modify the stability of the desynchronized unstable fixed point of (8). Two pronounced minima appear for stimulation periods of 8.1 and 4.0. Panel B compares the predicted (solid lines) and actual (colored dots) Floquet exponents for various combinations of stimulation frequency and amplitude. Panel C shows snapshots of the resulting periodic orbits induced by sinusoidal forcing with a period of 4.5 milliseconds. A bimodal distribution emerges that becomes more pronounced as the amplitude of forcing increases. Periodic orbits obtained using *A*≤3.5 *μA*/cm^2^ are unstable, the remaining orbits are stable.

To begin the analysis, the function *α*_1_(*T*) is determined numerically. This function characterizes the change of *λ*_1_ (the unstable eigenvalue) in response to perturbations. Results are shown in panel A of Fig. 2. Real(*α*_1_(*T*)) has two distinct local minima occurring at *T* ≈ 8.1 and 4.0 ms. Notice that the associated periods at which these minima occur are approximately equal to 2*π*/Imag(*λ*_1_) and 2*π*/Imag(*λ*_3_). This resonance can be explained by noting that the eigenvalues of the fixed point directly influence the magnitude of solutions that determine ${C}_{1}^{1}(\theta )$ from (44) which in turn corresponds to the magnitude of *α*_1_(*t*) (as illustrated in the Methods section).

Of particular interest here is how periodic stimulation can be used to desynchronize the synchronized stable periodic orbit of (8). While the peak in Real(*α*_1_(*T*)) at approximately *T* = 8.1 ms is large, stimulation at this frequency engenders resonance that results synchronous behavior. Instead, more focus will be given to the minimum occurring at around *T* = 4.0 ms. As illustrated in panel B of Fig. 2, applying sinusoidal stimulation ${I}_{{\rm{stim}}}(t)=A\sin (2\pi t/T)$, one finds good agreement between the numerically determined Floquet exponents (colored dots) and the values expected by (3) (solid lines). Particularly, (3) accurately predicts the stimulation magnitude required to stabilize the unstable fixed point, i.e., the value of *A* for which Real(*κ*_1_) crosses 0. Panel C shows a snapshot of the resulting periodic orbit induced by periodic sinusoidal stimulation with a period of 4.5 ms. The probability distribution becomes bimodal and the peaks of each mode grow with the stimulation amplitude.

Behavior of the Fokker-Planck distribution (8) is compared to the behavior of the full neural model (6) taking *N* = 1000 neurons. Stochastic simulations are performed using a second order accurate algorithm^52^ with a timestep of 0.005 ms. All model parameters are identical to those used to derive (8) except that *σ*_*c*_ is decreased by 37.5% so that the behavior can be investigated for small inputs. Synchronization is characterized by the Kuramoto order parameter^53^

12 $$R(t)=\left|\frac{1}{N}{\sum }_{k=1}^{N}{e}^{i{\theta }_{k}(t)}\right|.$$

**Figure 3:**
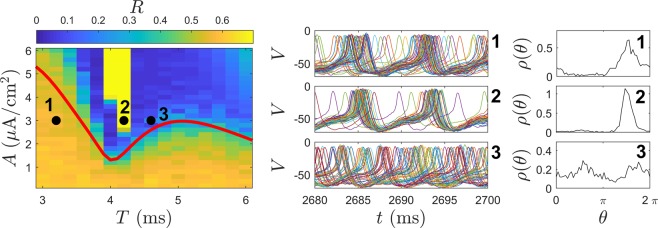
In the left panel, the average value of the Kuramoto order parameter (12) is shown for various combinations of stimulation frequency and amplitude after the transient behavior has died out. The red line gives a prediction for when desynchronization will occur based on the analysis of the underlying Fokker-Planck equation. Details of simulations using three different parameter sets represented with black dots are also shown. The middle panels show voltage traces for 50 representative neurons (in units of mV) over the course of about 2 periods. The right panels give a snapshots of the probability density inferred from the phase data.

In the above equation, *θ*_*k*_ = 0 is defined to be the moment that the *k*^th^ neuron spikes, and the phase *θ*_*k*_(*t*) is inferred at all other times using linear interpolation. *R*(*t*) = 1 when the neurons are completely synchronized and 0 for a uniform distribution. While the Kuramoto order parameter is not a perfect measurement of synchronization in all applications, it does work well here. The left panel of Fig. 3 shows the average order parameter calculated during 1000 ms after transient behavior has died out for various magnitudes and frequencies of sinusoidal perturbation applied to (6). Initial conditions are chosen so that the system is completely synchronized initially; additional simulations with different initial conditions do not yield qualitatively different results. The red curve shows a plot of $\sqrt{-{\rm{Real}}({\lambda }_{1})/{\rm{Real}}({\alpha }_{1}(T))}$. From (3), this curve represents a prediction of when desynchronization will occur through stabilization of the unstable fixed point assuming that Real(*λ*_1_) = 0.003, where *λ*_1_ is the unstable eigenvalue. Note that *λ*_1_ is not calculated numerically for (6), but that using this value provides a near perfect agreement for the observed threshold at which desynchronization occurs. Simulations corresponding to specific parameter sets are shown in the middle and right panels of Fig. 3. For parameter set 1, the stimulation amplitude is too low to stabilize the desynchronized fixed point. For stimulation with a period of 4.2 ms, desynchronization occurs once the amplitude reaches the expected threshold, but a secondary bifurcation results in strong synchronization illustrated with parameter set 2. Finally, parameter set 3 is slightly above the threshold which predicts desynchronization; the activity of the neurons is nearly incoherent, with two small amplitude peaks in the associated probability distribution.

### Conduction block in cardiac cells resulting from stabilization of a depolarized state through high-frequency stimulation

**Figure 4:**
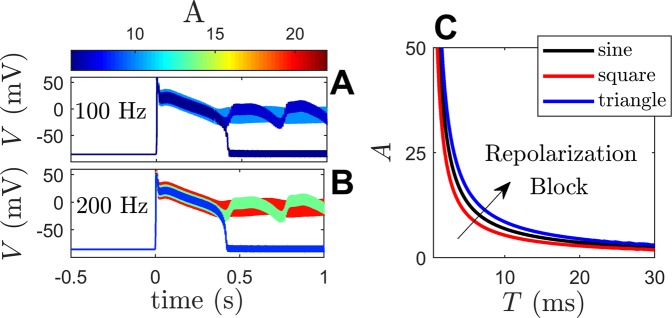
Panels A and B illustrate model behavior under the application of sinusoidal stimulation of various amplitudes and periods. In panel C, stimulation parameters to the right of the solid lines will produce repolarization block.

Here, the Shannon-Puglisi-Bers model^54^ for rabbit cardiomyocytes will be considered. This model describes the dynamical change of sodium, calcium, and transmembrane voltages within different compartments of a ventricular cardiomyocyte with the addition of several ionic buffers. The cellular dynamics will be considered first and will be followed by investigation 2-dimensional simulations. The general equations for this model are

13 $$\begin{array}{lll}\dot{V} & = & -{I}_{{\rm{ion}}}(w)+{I}_{{\rm{stim}}}(t)+{I}_{{\rm{pace}}}(t),\\ \dot{w} & = & {F}_{{\rm{aux}}}(V,w),\end{array}$$

where the *V* represents the voltage dynamics, *I*_ion_ gives the sum of all ionic currents, ${I}_{{\rm{stim}}}$ is a high-frequency external current, *I*_pace_ is an external pacing current, and *w* is a vector of 38 auxiliary variables which account for ion concentration dynamics, gating variables, ionic buffering and other model features. In (13), *t* is given in milliseconds. Model code was kindly provided by Dr. Puglisi and model parameters are identical to those used in^54^ except that the maximal conductances *g*_to_, f, *g*_to_, s and *g*_IKr_ are reduced by 50, 50, and 80 percent, respectively. This modification results in repolarization block for lower magnitude ionic currents and is implemented to better study the model behavior in a reduced framework, however, the qualitative results presented here do not change when the nominal parameter set is used instead. Pacing is applied at 1 second intervals by setting *I*_pace_ = 9.5 A/F when ${\rm{mod}}(t,1000) < 5$ ms and taking it to be zero otherwise. After pacing the model until steady state behavior is reached, pacing is removed and replaced with the high-frequency current ${I}_{{\rm{stim}}}(t)=A\sin (2\pi t/T)$. Figure 4 shows the general results for pacing with *T* = 10 ms and *T* = 5 ms (Panels A and B, respectively). When the magnitude of the external forcing becomes large enough, the cell will remain in a depolarized state indefinitely. Additionally as the period of stimulation is decreased, the magnitude of the stimulus required to achieve repolarization block increases; this general trend is illustrated in panel C of Fig. 4 for three different types of stimuli of varying amplitudes. Square wave input alternates back and forth between  ± *A* with a period T. Triangle wave input linearly ramps back and forth between  ± *A* with a period *T*.

**Figure 5:**
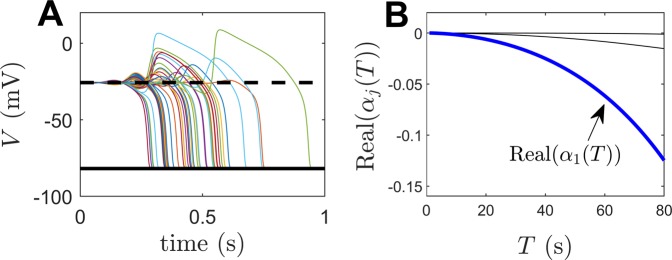
Simulation of a 14-variable reduced version of (13). Colored traces in Panel A show simulations with initial conditions near an unstable fixed point. The dashed (resp., solid) black line shows a simulation where the initial condition is taken to be the unstable (resp., stable) fixed point. Panel B shows the curves Real(*α*_*j*_(*T*)) from (3) calculated numerically corresponding to the first four eigenvalues of the unstable fixed point (here *α*_1_ and *α*_2_ are identical).

The general behavior illustrated in Fig. 4 can be understood more precisely as the stabilization of an underlying fixed point through periodic stimulation. To do so, a 14-variable analog will be used for analysis as described in the Methods section. This simplified model has two fixed points: one stable fixed point with *V* = −81.8 mV corresponding to the resting potential, and one unstable fixed point with *V* = −25.7 mV. Voltage traces of the reduced model for initial conditions near the unstable fixed point are shown in panel A of Fig. 5. The first four eigenvalues of the unstable fixed point are [*λ*_1_ = [0.015 + 0.034*i* *λ*_2_ = 0.015 − 0.034*i* *λ*_3_ = − 0.002 *λ*_4_ = −0.007], with curves Real(*α*_*j*_(*T*)) corresponding to each eigenvalue shown in panel B of Fig. 5. While this fixed point is indeed unstable, the eigenvalues with positive real part are not very large in magnitude. Additionally, Real(*α*_1_(*T*)) is negative and relatively large in magnitude suggesting that this system can be stabilized by periodic input. The function Real(*α*_1_(*T*)) increases with the period. This helps to explain the results from Panel C of Fig. 4 where repolarization block occurs for lower magnitude inputs when larger period stimuli are used. Arbitrarily large periods cannot be used, however. For periods larger than 80 ms, the state moves too far from the unstable fixed point and the asymptotic theory is invalidated.

**Figure 6:**
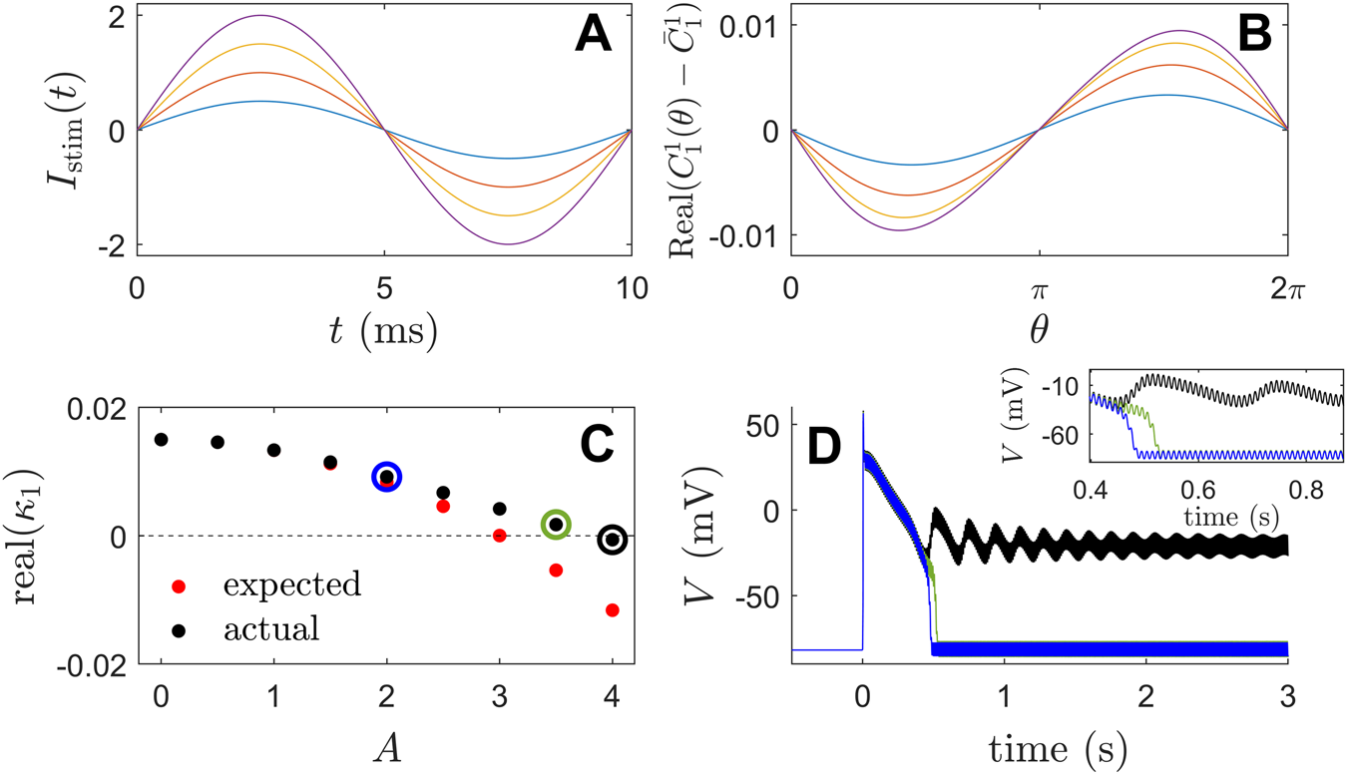
For the simplified 14-variable version of (13), panel A shows the magnitude of the applied stimulus. Plots in panel B of corresponding color show the real component of the mean-subtracted value of ${C}_{1}^{1}(\theta )$ for the periodic orbit induced by the external stimulus. Panel C shows the actual value of the principle Floquet exponent (black dots) and the expected values (red dots) based on the relationship (3). Colored circles correspond to voltage traces from Panel D with corresponding stimulation magnitudes.

  Figure 6 illustrates the change in the principle Floquet exponent, *κ*_1_, for the 14-variable model equations in response to periodic stimulation. In panel C of Fig. 6, the actual value of Real(*κ*_1_) is compared to the expected value calculated according to (3) using the numerically determined curve *α*_1_(*T*) with 100 Hz sinusoidal stimulation and amplitude *A*. The expected and actual Floquet exponents match well until *A* ≈ 3, at which point the actual value of Real(*κ*_1_) tends to be higher than the expected value. This can be explained by examining how the terms of the reduced functions (2) change as the stimulation amplitude grows. As explained in (23) from the Methods section, for a periodic orbit of (1) induced by external forcing *u*(*t*), changes in Floquet exponents from the forcing *u*(*t*) + *Δu*(*t*) can be characterized according to $\Delta {\kappa }_{i}=\frac{1}{T}{\int }_{0}^{T}{C}_{i}^{i}(\omega t)\Delta u(t)dt$. As shown in panels A and B of Fig. 6, ${\rm{Real}}({C}_{1}^{1}(\theta ))$ does not grow in proportion to ${I}_{{\rm{stim}}}(t)$ as the stimulus strength continues to increase. For this reason, the Floquet exponents do not change as fast as expected for larger values of *A*. Panel D shows the system behavior when periodic stimuli of varying strength are applied. Once the real component of the principle Floquet exponent becomes negative, the depolarized state becomes stable.

**Figure 7:**
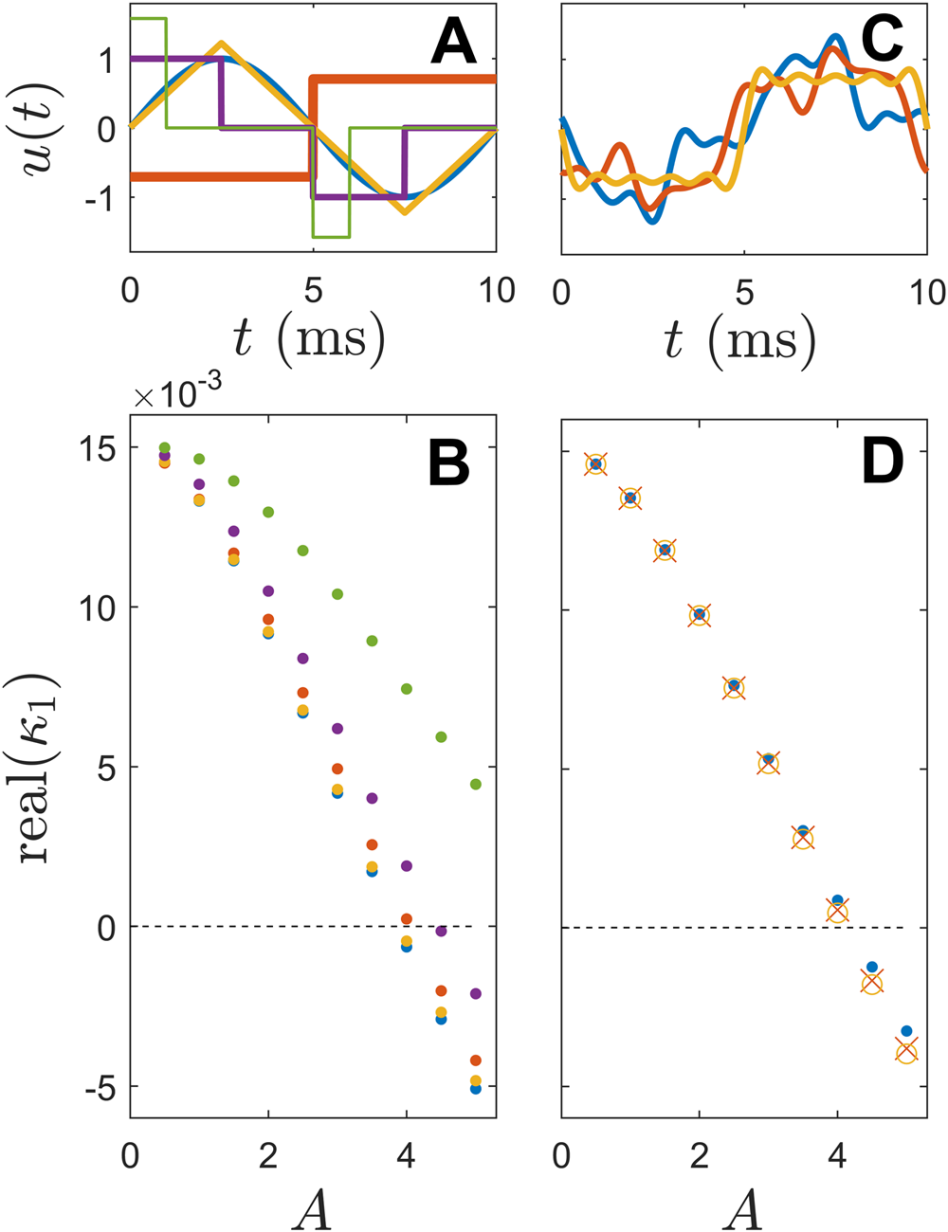
Comparisons between different stimulus shapes and resulting changes to Floquet exponents. In panel A, five different stimulus shapes (each with equal energy consumption) are shown. Each of these stimuli are multiplied by a given amplitude, *A*, and the resulting values of *κ*_1_ are shown in panel B. Colors of the individual dots correspond to the stimulus colors shown in the panel above. Similarly, three stimuli with identical ${a}_{k}^{2}+{b}_{k}^{2}$ for all *k* are shown in panel C. Markers of corresponding color in panel D illustrate that the resulting changes to *κ*_1_ are nearly identical for each stimulus. As expected, the sinusoidal forcing provides the maximum change in the Floquet multiplier at a given amplitude.

Next, the optimality of sinusoidal perturbations is assessed for the 14-variable model equations. In panel A of Fig. 7, the ability of five different stimuli with a period of 10 ms to stabilize the unstable fixed point is investigated. Each of these stimuli are normalized so that the overall energy consumption ${\int }_{0}^{T}{u}^{2}(t)dt$ among stimuli is identical. The resulting principle Floquet exponents are determined numerically for a periodic orbit induced by the stimulus ${I}_{{\rm{stim}}}(t)=Au(t)$. Results are shown in panel B. As expected, the pure sinusoid is most efficient for modifying the principle Floquet multiplier, and hence stabilizing the depolarized state. An interesting feature resulting from the relationship (3) is that for two different stimuli with identical values of ${a}_{k}^{2}+{b}_{k}^{2}$ for all *k* will yield the same change on the Floquet exponents. To illustrate this result, the periodic stimulus ${I}_{{\rm{stim}}}(t)=Au(t)$ is applied to the 14-variable model for 3 different values of *u* shown in panel C. Each *u*(*t*) have identical values of ${a}_{k}^{2}+{b}_{k}^{2}$. The resulting changes in the principle Floquet exponents are shown in Panel D confirming this expected behavior in the limit that the stimulus amplitude is small.

#### Conduction block under the application of a high-frequency electric field

In^10^ and^11^, conduction block imposed with high-frequency electric fields was investigated in the context of defibrillation. Here, using intuition from the previous section, high-frequency electric field stimulation is investigated with an eye towards the design of efficient strategies to impose conduction block on a large portion of tissue in order to eliminate spiral waves.

For simulations presented here, a bidomain model^55–57^ is considered with equations for the intracellular (*V*_*i*_) and extracellular potentials (*V*_*e*_) that follow the partial differential equation (PDE)

14 $$\begin{array}{lll}\nabla \cdot {\bar{\sigma }}_{i}\nabla {V}_{i} & = & \beta {I}_{m},\\ \nabla \cdot {\bar{\sigma }}_{e}\nabla {V}_{e} & = & -\beta {I}_{m},\\ {I}_{m} & = & {C}_{m}\frac{\partial {V}_{m}}{\partial t}+{I}_{{\rm{ion}}},\\ {V}_{m} & = & {V}_{i}-{V}_{e}.\end{array}$$

Above, ${\bar{\sigma }}_{i}$ and ${\bar{\sigma }}_{e}$ represent the intra and extracellular conductivity tensors, *β* = 1000 cm^−1^ is a constant which represents the surface area to volume ratio of the membrane, *V*_*m*_ gives the transmembrane voltage, and *C*_*m*_ = 1 *μ*F/*μ*cm^2^ is the membrane capacitance. Additionally, *I*_ion_ is a given cell’s ionic current density which is determined by the full Shannon-Puglisi-Bers model (13). Equation (14) is simulated on a square domain. For boundary conditions, it is assumed that there is no current flux across the intracellular domain (i.e., ${\bar{\sigma }}_{i}\nabla {V}_{i}\cdot \nu =0$) where, *ν* is a unit vector normal to the tissue boundary. Additionally, a time-varying electric flux is imposed along the left and right boundaries of the extracellular domain as would result from a high-frequency electric field; no flux boundary conditions are imposed along the top and bottom boundaries of the extracellular domain. Anisotropic conductivity tensors of the form

15 $${\bar{\sigma }}_{i}=\left[\begin{array}{lc}{g}_{ix}(x,y) & 0\\ 0 & {g}_{iy}(x,y)\end{array}\right],{\bar{\sigma }}_{e}=\left[\begin{array}{lc}{g}_{ex}(x,y) & 0\\ 0 & {g}_{ey}(x,y)\end{array}\right],$$

are used. In most locations, *g*_*ex*_, *g*_*ey*_, *g*_*ix*_, and *g*_*iy*_ are taken to be 0.8, 2.0 0.2, and 2.0 mS/cm, respectively. The anisotropy ratios are the same as the nominal values reported in^58^. As in^59^ and^60^, *g*_*ix*_ is set to zero in some locations to represent insulating plaque between myocardial fibers. The removal of these gap junctions results in the formation of virtual electrodes when an extracellular voltage gradient is applied (described below). In simulations, 250 sets of gap junctions are removed randomly with a minimum, maximum, and average length of 1.41, 3.96, and 2.69 millimeters along the principle fiber direction. Simulations of (14) are performed with an operator splitting scheme (detailed in equations (14) and (15) of ^61^) whereby the transmembrane voltage is found each iteration with a forward Euler step and this information is subsequently used to update the extracellular voltage by solving a large linear system of equations. Simulations are performed on a 200 × 200 grid with a spatial and temporal discretization of 283 *μ*m and 0.007, respectively.

**Figure 8:**
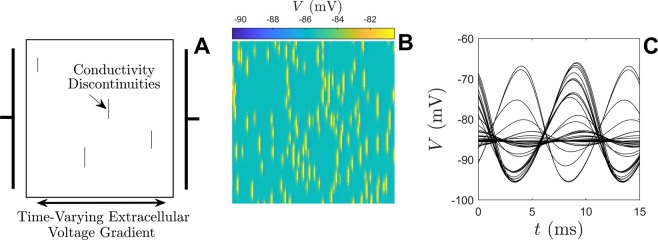
Panel A shows a schematic of the 2D bidomain model domain from (14). Black lines show a subset of the extracellular gap junctions that have been removed representing insulating plaque between myocardial fibers. The resulting interfaces between extracellular conductivities create virtual electrodes that act as activation sites when an extracellular voltage gradient is applied. Panel B gives a snapshot in time of the transmembrane voltage when a small amplitude alternating current is applied highlighting the locations of virtual electrodes. Figure C shows a representative set of voltage traces during the application of this alternating electric field. Cells that are closest to the virtual electrodes receive the largest influence from the extracellular voltage gradient. Cells that are far away from the virtual electrodes are not strongly influenced.

In all simulations electric field gradients are applied transverse to the principle fiber direction (horizontally) as illustrated in panel A of Fig. 8. In response to an extracellular electric field gradient, localized changes in transmembrane potential occur at the interfaces between locations with different conductivities. These activation sites are commonly referred to as secondary sources or virtual electrodes^60,62^. The virtual electrodes are illustrated in Fig. 8 where a 100 Hz alternating electric field with maximum strength of 1.76 V/cm is applied when all cells are initially quiescent. Subthreshold oscillations in the voltage can be observed near the virtual electrodes in response to the alternating voltage field. Panel B shows a snapshot of this behavior in time and panel C shows voltage traces for a representative sampling of cells in the tissue. Locations near the virtual electrodes display the largest oscillations in transmembrane voltage amplitude while locations far from these electrodes are nearly unaffected by the extracellular voltage field. Additionally, the influence of each virtual electrode depends on the size of its associated conductivity discontinuity. A detailed description of virtual electrodes in computational models and experimental preparations can be found in^60^.

**Figure 9:**
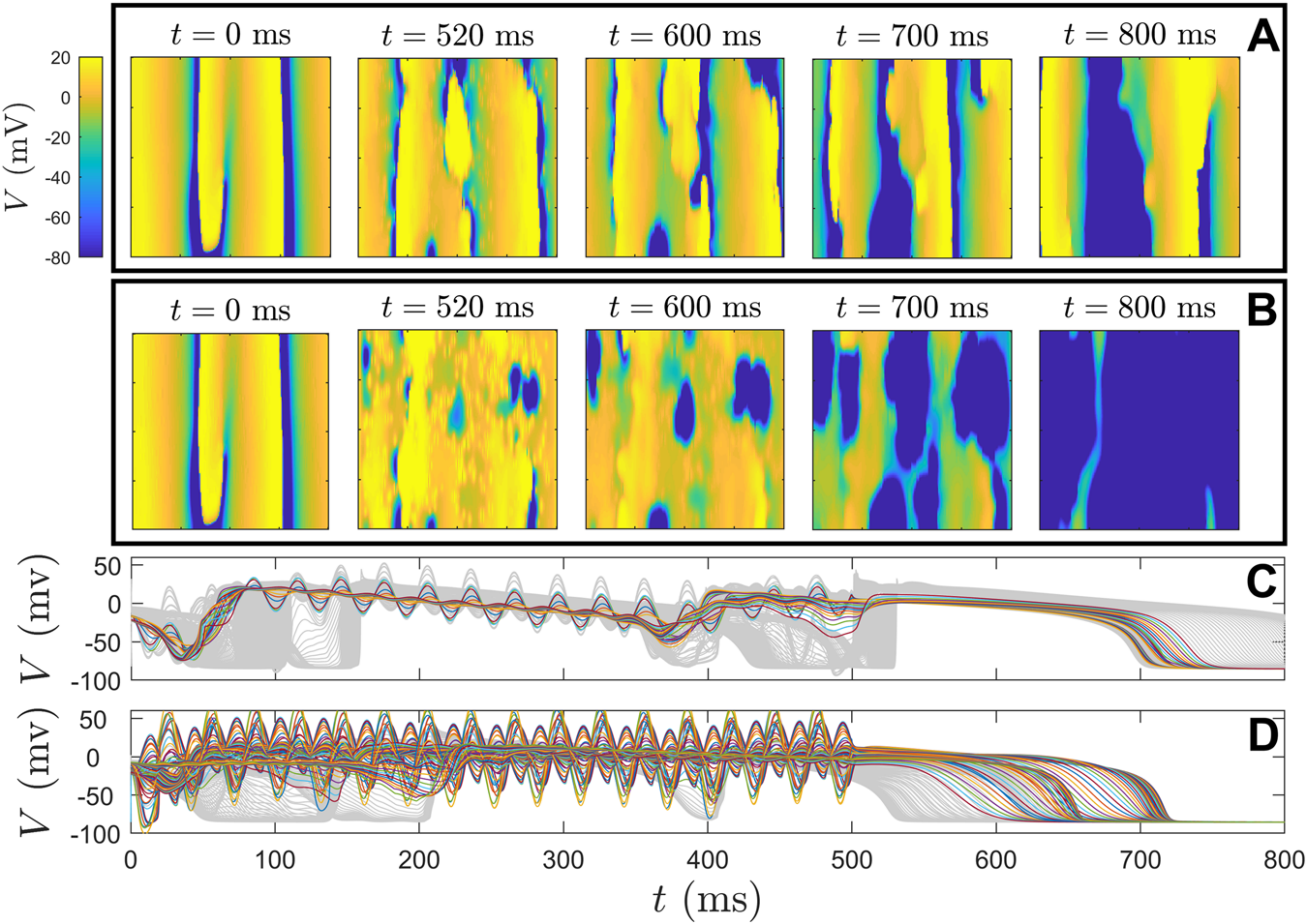
Elimination of spiral waves on a 2-dimensional domain with an alternating current electric field. In panel A (resp., B) a 33 Hz alternating electric field of 1.76 V/cm (resp., 5.29 V/cm) lasting 500 ms is applied to (14). Initial conditions in both simulations are identical with a single spiral wave present at *t* = 0. Panels C and D show 200 individual cell voltage traces corresponding to simulations from panels A and B, respectively. These cells are taken from the vertical centerline of the square domain. Note that the cells closest to the virtual electrodes are influenced most strongly by the applied extracellular voltage gradient. In panels C and D, cells that remain depolarized during the entire application of the alternating electric field are plotted in color. As expected, larger magnitude voltage fields result in increased transmembrane current flow near the tissue heterogeneities. The number of cells that experience conduction block (i.e., with a stabilized depolarized state) increases as the magnitude of the extracellular stimulation increases. This effectively splits the larger domain into smaller subdomains so that the spiral can be absorbed.

In the simulations to follow, the electric field will be investigated in terms of its ability to incite conduction block throughout the tissue. Viewing the results to follow in the context of the theory from the previous section, it will be assumed that repolarization block occurs near virtual electrodes when the applied electric field is strong enough; consequently this inhibits spiral wave propagation. Note that the application of forcing here is different than in the neurological application from the previous section. In the neurological application, a transmembrane current is applied to all cells identically. In the application considered here, an extracellular current is applied to the domain and its influence on an individual cell depends on the relative location of the virtual electrodes. A single spiral wave is initiated in (14) with a premature stimulus after pacing to steady state at a rate of 1 hz. These spiral waves are stable and persist indefinitely when no extracellular voltage gradient is applied. Figure 9 shows the resulting dynamical behavior when a sinusoidal, 33 Hz alternating electrical field is applied to the bidomain model (14). In panel A, the strength of the electric field is low (1.76 V/cm); the trajectory of the spiral is not significantly influenced and persists after the electric field is removed. The field strength is three times larger in panel B (5.29 V/cm) resulting in more conduction block throughout the domain. After the alternating field is removed, all spirals are extinguished. Panel C (resp., D) shows sample transmembrane voltage traces from individual cells from the simulations in panel A (resp., B). Colored traces highlight cells that remain depolarized throughout the entire application of the alternating electric field. These numerical simulations are repeated over multiple trials to test the capabilities of 100 Hz alternating current electric field on its ability to to eliminate spiral waves.

**Figure 10:**
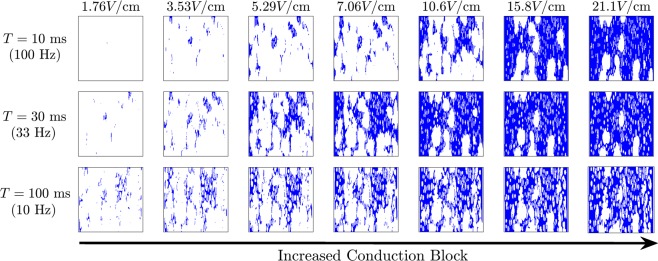
Under the application of a high-frequency external voltage field, locations that experience conduction block are shown in blue. For low amplitude stimulation, conduction block only occurs near large discontinuities in internal conductance. As the stimulation amplitude increases, conduction block becomes more prevalent partitioning the larger domain into disconnected subdomains that ultimately absorb spirals. In general, higher frequencies require a larger stimulation amplitude to elicit conduction block as predicted from the shape of *α*_1_(*T*) from Figure 5. For periods larger than about 80 ms, the asymptotic theory used to derive Equation (3) begins to break down. For each frequency shown here, electric fields with larger strength than those shown do not result in more conduction block.

Conduction block is investigated in greater detail for varying electric field strengths and frequencies. Each panel from Fig. 10 shows the result of a 500 ms duration alternating current application with initial conditions taken to be identical to those from Fig. 9. For a cell at a given location on the domain, if the transmembrane voltage remains above  − 30 mV from *t* = 50 ms to *t* = 500 ms, the corresponding location is color coded blue in the plots in Fig. 10 (note that in the absence of an electric field cells never remain depolarized past  − 30 mV for longer than 410 ms. As illustrated in Fig. 10, the proportion of cells experiencing conduction block increases with electric field strength. Additionally, larger electric field strengths are necessary to induce conduction block for higher frequency stimuli which is consistant with the shape of the *α*_1_(*T*) curve from Fig. 5. Also notice that the 10 Hz frequency does not do much better than the 33 Hz stimulation; when the slow 10 Hz forcing is applied, the state moves too far from the unstable fixed point thereby invalidating the assumptions used to derive (3).

Results from Fig. 10 can help to explain the propensity for high-frequency electrical current to extinguish spiral waves: for a specific combination of frequency and electric field magnitude, if enough cells experience conduction block the domain is effectively split into multiple, smaller subdomains with absorbing boundary conditions. If these subdomains are small enough, any spirals will ultimately be absorbed while the alternating electric field is applied resulting in quiescence when the field is removed. This mechanism is similar to the one suggested by^63^ and agrees with experimental findings from^10^ and^11^. This interpretation is supported with additional simulations to test the ability of 100Hz an alternating electric current field to eliminate spiral waves. Using field strengths of 21.1 V/cm (resp. 7.06 V/cm), over 11 trials with a single spiral wave present, the stimulation is successful at eliminating the spiral within 500 ms after stimulation ends 11 (resp. 4) times. While the sample size is small, Wilson score intervals at 95 percent confidence place the true success probability between 0.74 and 1 for the stronger stimulation and between 0.15 and 0.64 for the weaker stimulation denoting a significant difference in success rates for the different stimulation magnitudes.

## Discussion

HFES has shown tremendous potential as a treatment for a wide variety of neurological disorders^1,2,4–6^. Emerging evidence also suggests that HFES could be used as an intervention for cardiac arrest^10–12^ and could be used to develop new strategies for pain management^8,9^. Despite its wide study in various biological applications, the underlying physiological mechanisms responsible for the therapeutic benefit of HFES are often difficult to interpret. In this work, detailed investigation of two different, medically relevant applications suggests a common dynamical mechanism underlying the therapeutic benefit of HFES. Specifically, theoretical analysis and numerical modeling illustrate that appropriately designed electrical stimuli may alter qualitative dynamical behavior by stabilizing unstable fixed points of a high-dimensional nonlinear dynamical system. This hypothesis represents a unifying mechanism which could be used as a starting point to better understand the therapeutic benefit of deep brain stimulation as a treatment for neurological diseases such as Parkinson’s disease and could be useful for designing high-frequency defibrillation waveforms. Given that the applications presented here are completely different in scope, it may be the case that stabilization of an underlying fixed point could explain observed behaviors in other biological applications where HFES has been shown to be effective.

### Theoretical analysis

Novel theoretical analysis provided in this work uses a nonlinear isostable reduction framework^24,25^ to characterize the shift in Floquet exponents resulting from periodic stimulation. As part of this analysis, a nonlinear reduction framework is necessary because the linear terms of the reduction average to zero. Compared to the methods employed in other studies to investigate the effects high-frequency stimulation^12,17,27,64^, the preliminary transformation to isostable coordinates used here allows one to directly study the change in specific Floquet multipliers and can be straightforwardly implemented in arbitrarily high-dimensional nonlinear systems. Further analysis reveals the unexpected result that a purely sinusoidal waveform is energy optimal for stabilizing an unstable fixed point with periodic stimulation for all dynamical systems with one unstable eigenvalue (or one complex-conjugate pair of unstable eigenvalues). Of course, energy is not the only important consideration when designing stimulation for biological systems and other cost functionals could be considered that account for other important features such as limiting Faradaic charge transfer^30,65^. For certain systems, the theoretical analysis presented here can be used to provide fundamental limits on the ability of periodic stimulation to stabilize an unstable fixed point. For example, if the function *α*_1_(*T*) from (3) associated with an unstable fixed point is strictly positive, periodic stimulation will not be able to stabilize the fixed point. In this case, closed-loop strategies would be necessary. It is worth mentioning that the present theoretical results only apply to stabilization of a weakly unstable fixed point. Other control objectives will yield different results. For instance^30^, discusses the tradeoff between action potential initiation, tissue damage, and electrode corrosion for various types of waveforms used in deep brain stimulation. Energy optimal waveforms for neural action potential initiation were investigated in^66^ with resulting waveforms that resembled Gaussian curves. Optimal waveforms for entrainment of oscillators that minimize the time required for entrainment^34^ or the absolute value of applied charge^35^ have also been investigated. Additionally^67^, considered different waveforms for engendering desynchronization in adaptive deep brain stimulation algorithms. In general, different waveforms will be obtained when considering different objectives and when using different cost functions for optimization.

A significant limitation of the theoretical analysis presented here is that the Floquet exponent predictions from (3) are valid in the limit that the external stimulation is small. As such, modifying the stability of an unstable fixed point requires that any unstable eigenvalues must have a small magnitude real component, otherwise the prediction for the resulting Floquet exponents will break down before a stabilizing bifurcation can be reached. It may be of interest to develop strategies for predicting changes to Floquet exponents that go beyond second order accuracy to extend the applicability of the methods presented here for larger magnitude inputs. This will be the subject of future investigation.

### Desynchronization of neural populations

In the first application considered in this work, desynchronization of a large population of pathologically synchronized oscillators through the application of external current stimulation is considered. A similar problem has been investigated previously using the dynamics of uncoupled neurons as a starting point. For instance^41^, identifies specific frequencies which result in a positive Lyapunov exponent and^42^ investigates the tendency of a large population of neurons to be separated into equal clusters in the presence of low-intensity noise and periodic stimulation. While both of these strategies provide novel insight about underlying neural dynamics, they both assume that the neurons are uncoupled making it difficult to examine interplay between the external stimulation and the coupling that causes the synchronization in the first place. Conversely, in the present work computational modeling results suggest that stimulation at specific frequencies can stabilize an underlying unstable fixed point of the Fokker-Planck representation of the probability distribution dynamics (8) which subsequently results in a desynchronized distribution. Numerical results indicate that certain stimulation frequencies are more energy optimal at modifying the principle Floquet exponents. This result is consistent with an experimentally observed frequency dependence on the energy efficiency of deep brain stimulation^68,69^ with 130 Hz stimulation usually being used to maximize the tradeoff between clinical effectiveness and overall power consumption. Furthermore, computational modeling results presented here suggest a narrow window of amplitudes that result in desynchronization for a particular stimulation frequency: the stimulation must be large enough to stabilize the underlying fixed point but not too large that it causes additional bifurcations that result in synchronization.

The results presented here should be interpreted with caution. The focus here considers the stabilization of an underlying fixed point that represents a desynchronized state. However, for a population of neurons that are initially synchronized, merely stabilizing a desynchronized state does not guarantee that this state will ultimately be reached (i.e., a given initial condition may not be in the basin of attraction of the stable attractor). In different populations of coupled oscillators there may be intermediate periodic, quasiperiodic, or chimera states (see^37^ for example) that would preclude desynchronization. Additionally, stable multimodal distributions have the tendency to emerge when stronger periodic forcing is administered; this is observed in Panel C of Fig. 2 as the magnitude of forcing becomes stronger. Finally, the results presented here are only valid in the limit of weak periodic stimulation. This weak stimulation assumption performs well for predicting the boundary between high and low order parameters in the left panel of Fig. 3 (red curve). However, this theory cannot explain the secondary bifurcation that leads to synchronization when the magnitude of forcing is increased, for instance, as observed in the second parameter set from Fig. 3. These issues will be addressed in future work.

While the model considered here provides insight into the dynamical behavior of periodically stimulated populations of tonically firing neurons, limitations of this component of the computational modeling study are numerous. Foremost, the numerical model considered uses lumped conductance based models that do not account for the complicated interplay between the electric field generated by an external probe and the spatial positioning of a neuron’s soma, axons and dendrites^13,14^. These factors would need to be considered to better understand how these results can be applied to the design of better DBS stimulation protocols. Additionally, these results only consider the behavior of a single population of coupled neurons and more detailed modeling would be needed to identify the effect of a desynchronizing stimulation on the overall network circuit. Heterogeneity in neural properties has not been considered in this study. In future work, it may be possible to explicitly account for population heterogeneity in neural phase response curves^70^ and nominal firing rates and incorporate this information into the Fokker-Planck representation from (8). Finally, other compelling evidence exists that suggests that during clinically effective DBS, neural action potentials become entrained to the external stimulation^71–73^, a feature that is not directly addressed here. Nevertheless, similar to the results from^42^, during effective desynchronizing frequencies, the neural probability distribution becomes bimodal illustrating that entrainment and desynchronization are not necessarily mutually exclusive in these models.

### Cardiac conduction block

In the second application considered in this work, the ability of periodic electrical stimulation to induce conduction block is investigated. Theoretical and computational results presented here are consistent with experimental and computational results from^10^ and^11^, specifically that the imposition of conduction block in cardiac cells requires electric field strengths that are directly proportional to the frequency of the applied electric field. Theoretical analysis of low order models in the high-frequency limit of forcing has identified similar patterns^12,17,18^. In^12^, a reduced order phenomenological model was investigated with results that suggest a gradual transition between membrane potential elevation and periodic stimulation intensity. In this work the exact opposite conclusion is reached using a more detailed computational model: conduction block emerges as the result of a bifurcation that leads to a dynamically stable elevated membrane potential. The mechanisms responsible for conduction block in response to HFES have important implications to the design of alternative high-frequency defibrillation waveforms. As such, further experimental investigation is required to identify which proposed mechanism is more accurately reflected by living cardiomyocytes.

There are many limitations to the computational results presented here. While the model considered in this work^54^ contains a physiologically accurate representation of ionic currents, ionic pumps and calcium signaling, modifications to the ionic buffering calcium release from the ryanodine receptors (as illustrated in the Methods section) were necessary to perform the theoretical analysis. Despite this modification, the full model equations displayed qualitatively similar behaviors to the modified model. Furthermore, the bidomain simulations considered in this work were performed on a two-dimensional square domain. This is a drastic oversimplification and simulations performed here do not take realistic three-dimensional heart geometries, fiber architecture or spatial heterogeneities^55,74^ into account. The primary purpose of this study is to highlight a potential mechanism for cardiac conduction block observed during the application of a high-frequency electric field and is not meant to be an exhaustive study of the potential for alternating current waveforms to be used as an alternative approach to defibrillation. Rather, alongside the results from^10^ and^11^ the simulations presented here suggest that continued investigation of alternating current defibrillation methods would be warranted.

## Methods

### Second order accurate averaging methods

Consider an externally forced nonlinear ordinary differential equation

16 $$\dot{x}=F(x)+\epsilon g(t),$$

with $x\in {{\mathbb{R}}}^{n}$, *F* gives the nominal dynamics, and $g(t)={\left[u(t)0\ldots 0\right]}^{T}$ is a *T*-periodic, exogenous input, and 0≤*ϵ* ≪ 1. Further, suppose that $\bar{\rm{u}}={\int }_{0}^{T}u(t)dt=0$, i.e., its averaged value is zero. Let *x*_0_ be a fixed point of *F* with eigenvalues *λ*_1_, …, *λ*_*n*_ ordered so that ${\rm{Re}}({\lambda }_{i})\le {\rm{Re}}({\lambda }_{i+1})$. The goal of this analysis is to understand how periodic perturbations can influence the stability of *x*_0_. Dynamical averaging is often used to approach this type of problem. As an example of this approach, consider a linear approximation of (16) near the fixed point at *x*_0_

17 $$\Delta \dot{x}=J\Delta x+\epsilon g(t),$$

here, Δ*x* ≡ *x* − *x*_0_ and is assumed to be ${\mathcal{O}}(\epsilon )$ and *J* is the Jacobian of the vector field *F* evaluated at the fixed point. Equation (17) is *T*-periodic and in the general form

18 $$\dot{x}=\epsilon Q(x,t),$$

so that averaging techniques can be used^75,76^ resulting in a function of the form

19 $$\dot{X}=JX+\epsilon  {\bar{g}} =JX.$$

Here, ${\bar{g}} =\frac{1}{T}{\int }_{0}^{T}g(t)dt=0$. Fixed points of (19) correspond to periodic solutions of (17) with the same stability^75,76^. Because (19) is no different from the linearization of $\dot{x}=F(x)$, first order accurate averaging strategies are not sufficient to characterize a change in stability and higher order accurate effects must be considered.

Unfortunately employing higher order accurate averaging methods tends to be less analytically tractable and more computationally intensive for high dimensional models. As shown in^27^, to leading order *ϵ*^2^ the logarithm of the average of the Monodromy map of the resulting periodic orbit induced by the perturbation *g*(*t*) can be approximated as^27^

20 $$Y=\epsilon \bar{Q}+\frac{{\epsilon }^{2}}{2T}{\int }_{0}^{T}[{\int }_{0}^{t}Q(x,\tau )d\tau ,Q(x,t)]dt,$$

where [ ⋅ , ⋅ ] denotes Jacobi-Lie bracket and $\bar{Q}=\frac{1}{T}{\int }_{0}^{t}Q(x,\tau )d\tau$. Related averaging results are also shown in^75^. The second-order accurate approach from (20) has been used, for instance, in^64^ to asses stability of hovering insects and in^27^ to design control strategies for biomimetic locomotion. While these averaging strategies can be used to asses the change in the Floquet exponents to second-order accuracy, (and hence asses changes in stability), it is difficult to use this approach to understand a perturbation’s direct influence on specific Floquet exponents; as the size and complexity of the systems under consideration grows, computation of Floquet exponents using (20) can only be accomplished numerically.

### Phase-amplitude reduction using isostable Coordinates

In this work, an alternative framework is applied which uses isostable coordinates to characterize direct changes to desired Floquet exponents. While in principle, this can be used to directly manipulate any Floquet exponent, this work will focus on dynamical systems that are close to a bifurcation, i.e., with Floquet exponents that are close to zero. A significant advantage over other second-order accurate averaging techniques is that the method presented here yields a relatively simple relation for the change in a specific Floquet exponent induced by a periodic perturbation which can be used for design purposes. To begin, Eq. (16) can be written in a form with an autonomous periodic orbit by redefining the time variable according to (cf.,^77^)

21 $$\dot{y}=\frac{d}{dt}\left[\begin{array}{c}x\\ s\end{array}\right]=\left[\begin{array}{c}F(x)+\epsilon g(s)\\ 1\end{array}\right]={F}_{a}(y),$$

where $$y\in {{\mathbb{R}}}^{n}\times {{\mathbb{R}}}^{1}$$.

From this perspective for a periodic solution to (21), one can view the behavior in terms of phase and isostable coordinates,

22 $$\begin{array}{lll}\dot{\theta } & = & \omega +\left(Z(\theta )+{\sum }_{k=1}^{n}{\psi }_{k}{B}^{k}(\theta )\right)\Delta u(s),\\ {\dot{\psi }}_{j} & = & {\kappa }_{j}{\psi }_{j}+\left({I}_{j}(\theta )+\mathop{\sum }\limits_{k=1}^{n}{\psi }_{k}{C}_{j}^{k}(\theta )\right)\Delta u(s).\\ j & = & 1,\ldots ,n-1\end{array}$$

Here, *θ* is the phase of oscillation which gives a sense of the location along a stable periodic solution, *ψ*_*j*_ is the *j*^th^ isostable coordinate which gives a sense of the distance from the periodic orbit in a particular basis, *ω* = 2*π*/*T* is the natural frequency, *κ*_*j*_ is the Floquet exponent associated with the *j*^th^ isostable coordinate, *Z*(*θ*) and *I*(*θ*) are the phase and isostable response curves, respectively, which give the first order accurate dynamics, and *B*^*k*^(*θ*) and ${C}_{j}^{k}(\theta )$ provide second-order accurate corrections. Finally, recalling that $$g(s)={\left[\begin{array}{cccc}u(s) & 0 & \ldots  & 0\end{array}\right]}^{T}$$, *Δu*(*s*) represents the deviation from the nominal input *u*(*s*). Information about the derivation of (22) and strategies for the numerical calculation of all necessary terms of the reduction can be found in both^24^ and^25^. In the specific situation considered in this work, as a consequence of the periodic orbit in (22) being induced by the periodic forcing, phase and time are related according to *θ*(*s*) = *ωs*.

In^78^, it was shown that for some Δ*u*(*s*), changes to the Floquet exponents Δ*κ*_*j*_ ≡ *κ*_*j*_[Δ*u*(*s*)] − *κ*_*j*_[0] are well approximated by

23 $$\Delta {\kappa }_{j}=\frac{1}{T}{\int }_{0}^{T}{C}_{j}^{j}(\omega s)\Delta u(s)ds.$$

Equation (23) will be used as a starting point to characterize the nonlinear change in Floquet exponents in response to a periodic input.

### General structure of the functions of the isostable reduction

It will be shown that for a given system (21), the function $\frac{1}{T}{\int }_{0}^{T}{C}_{j}^{j}(\omega s)\Delta u(s)ds$ is proportional to *ϵ*, the magnitude of the external forcing. Once this is established, equation (23) can be used to derive the relationship (3). Additionally, equations will be provided to estimate the *α*_*j*_(*T*/*k*) from (3) based on the nonlinear terms of the phase-isostable reduction (22) and the input *u*(*t*) itself.

To proceed, for a given *T*-periodic orbit *y*^*γ*^(*t*) of (21), the terms of the first order accurate part of the reduction (22) can be found by computing the periodic solutions to the following equations^79,80^:

24 $$\frac{d{\mathcal{Z}}(\theta (t))}{dt}=-{J}_{{y}^{\gamma }(t)}^{T}{\mathcal{Z}}(\theta (t)),$$

25 $$\frac{d{{\mathcal{I}}}_{j}(\theta (t))}{dt}=({\kappa }_{j}{\mathbb{I}}-{J}_{{y}^{\gamma }(t)}^{T}){{\mathcal{I}}}_{j}(\theta (t)),$$

where ${J}_{{y}^{\gamma }(t)}$ represents the Jacobian of the the vector field *F*_*a*_ evaluated at *y*^*γ*^(*t*), *κ*_*j*_ is the Floquet exponent associated with the *ψ*_*j*_ isostable coordinate, ^*T*^ denotes the transpose of a matrix, ${\mathbb{I}}$ is the identity matrix. Equations (24) and (25) are subject to the normalizing conditions ${F}_{a}{(y(\theta ))}^{T}{\mathcal{Z}}(\theta )=\omega$ and ${v}_{j}^{T}{{\mathcal{I}}}_{j}(\theta )=1$ where *v*_*j*_ is an eigenvector of the fundamental matrix associated with the eigenvalue ${\lambda }_{j}=\exp ({\kappa }_{j}T)$. Note here that *Z*(*θ*) (resp., *I*_*j*_(*θ*)) from (22) is the first component of ${\mathcal{Z}}(\theta )\in {{\mathbb{R}}}^{n+1}$ (resp., ${{\mathcal{I}}}_{j}(\theta )\in {{\mathbb{R}}}^{n+1}$). The second order accurate components of (22) can be determined by finding periodic solutions to the equations^25^

26 $$\frac{d{{\mathcal{B}}}^{k}(\theta (t))}{dt}=-\mathop{\sum }\limits_{i=1}^{n+1}\left({{\mathcal{Z}}}^{i}(\theta (t)){H}_{i,{y}^{\gamma }(t)}{p}_{k}(\theta (t))\right)-({J}_{{y}^{\gamma }(t)}^{T}+{\kappa }_{k}{\mathbb{I}}){{\mathcal{B}}}^{k}(\theta (t)),$$

27 $$\frac{d{{\mathcal{C}}}_{j}^{k}(\theta (t))}{dt}=-\mathop{\sum }\limits_{i=1}^{n+1}\left({{\mathcal{I}}}_{j}^{i}(\theta (t)){H}_{i,{y}^{\gamma }(t)}{p}_{k}(\theta (t))\right)-({J}_{{y}^{\gamma }(t)}^{T}+({\kappa }_{k}-{\kappa }_{j}){\mathbb{I}}){{\mathcal{C}}}_{j}^{k}(\theta (t)),$$

where ${{\mathcal{Z}}}^{i}(\theta )\equiv \partial \theta$/∂*y*_*i*_ and ${{\mathcal{I}}}_{j}^{i}(\theta )\equiv \partial {\psi }_{j}$/∂*y*_*i*_, both evaluated on the periodic orbit (in other words, these are the *i*^th^ components PRC and IRC, respectively), ${H}_{i,{y}^{\gamma }(t)}$ is the Hessian of the *i*^th^ element of *F*_*a*_ evaluated at *y*^*γ*^(*t*), and *p*_*k*_(*θ*(*t*)) is the eigenfunction associated with the Floquet expansion of the solution at locations close to the periodic orbit. In the above equations, *B*^*k*^(*θ*) (resp. ${C}_{j}^{k}(\theta )$) from (22) gives the first component of ${{\mathcal{B}}}^{k}(\theta )\in {{\mathbb{R}}}^{n+1}$ (resp., ${{\mathcal{C}}}_{j}^{k}(\theta )\in {{\mathbb{R}}}^{n+1}$).

To proceed, consider the case where *ϵ* = 0. The resulting attractor of (21) is the fixed point ${y}_{0}^{\gamma }$, and can still technically be viewed as a *T*-periodic orbit. Define ${{\mathcal{I}}}_{j}^{\epsilon }(\theta )$ and ${{\mathcal{C}}}_{j}^{j,\epsilon }(\theta )$ as appropriately normalized solutions of (25) and (27) for the equation (21) for the periodic orbit *y*^*γ*^(*t*) obtained using the stimulus *ϵg*(*t*). In the case where *ϵ* = 0, solutions of (25) and (27) are constant in time (since the attractor is a fixed point). Additionally, when *ϵ* = 0, *κ*_*j*_ = *λ*_*j*_.

When *ϵ* is not zero, consider the resulting deviation from the fixed point: $\Delta y(t)\equiv {y}_{\epsilon }^{\gamma }(t)-{y}_{0}^{\gamma }$, where ${y}_{\epsilon }^{\gamma }(t)$ denotes the periodic orbit for a given value of *ϵ*. For the moment, it will be assumed that the input *u*(*t*) is purely sinusoidal, i.e., $u(t)=\sin (\omega t+\phi )$ where *ϕ* is some phase shift. This assumption will be relaxed later. From linear systems theory^81^, to leading order *ϵ*, *Δy*(*t*) is the steady state solution to the periodically forced linearized system response from (17) and can be written as

28 $$\Delta y(t)=\epsilon {\left[\begin{array}{cccc}{x}_{1}^{ss}(t) & \ldots  & {x}_{n}^{ss}(t) & 0\end{array}\right]}^{T},$$

where ${x}_{j}^{ss}(t)=| {g}_{j}(i\omega )| \sin (\omega t+\angle {g}_{j}(i\omega )+\phi )$, with *g*_*j*_(*w*) denoting the Laplace transform of the input *u*(*w*) to state *j* (for this example, *g*_*j*_(*w*) is the *i*^th^ row and first column of the matrix ${(w{\mathbb{I}}-{J}_{{y}_{0}^{\gamma }})}^{-1}$), and ∣ ⋅ ∣ and *∠*( ⋅ ) denote the magnitude and argument, respectively.

The goal here is to identify how *p*_*j*_(*θ*), ${{\mathcal{I}}}_{j}(\theta )$, and ultimately ${{\mathcal{C}}}_{j}^{j}(\theta )$ from (22) change with increasing *ϵ*. The function *p*_*k*_(*θ*(*t*)) will be considered first. To proceed, let $\Delta {y}_{2}(t)=y(t)-{y}_{\epsilon }^{\gamma }(t)$ denote the distance from the periodic orbit. To leading order *ϵ*, using local linearization one can write

29 $$\Delta {\dot{y}}_{2}(t)={J}_{{y}_{\epsilon }^{\gamma }(t)}\Delta {y}_{2}(t)+{\mathcal{O}}({\epsilon }^{2}).$$

Recalling that ${y}_{\epsilon }^{\gamma }(t)=\Delta y(t)+{y}_{0}^{\gamma }$, one can expand the Jacobian from (29) and rewrite to leading order in *ϵ* as

30 $$\Delta {\dot{y}}_{2}(t)=\left({J}_{{y}_{0}^{\gamma }}+\Delta {J}_{{y}_{\epsilon }^{\gamma }(t)}\right)\Delta {y}_{2}(t),$$

where

31 $$\Delta {J}_{{y}_{\epsilon }^{\gamma }(t)}\equiv \left[\begin{array}{cccc}{H}_{1,{y}_{0}^{\gamma }}\Delta y(t) & \ldots \  & {H}_{n,{y}_{0}^{\gamma }}\Delta y(t) & 0\end{array}\right]$$

characterizes how the Jacobian changes near the fixed point and 0 corresponds to an appropriately sized vector of zeros (since the Hessian of the time-like variable dynamics is zero). Floquet theory^26^ states that solutions of (30) have the general structure

32 $$\Delta {y}_{2}(t)=\mathop{\sum }\limits_{k=1}^{n}{c}_{k}\exp \left(\left({\kappa }_{k}+\Delta {\kappa }_{k}\right)t\right){p}_{k}(\theta (t)),$$

where Δ*κ*_*k*_ is the change in Floquet exponent induced by the forcing and *p*_*k*_(*θ*) is the same function from the right hand sides of (26) and (27). Recall that Δ*κ*_*k*_ is an ${\mathcal{O}}({\epsilon }^{2})$ term and consider a specific solution of (30) written in the form (32) with *c*_*j*_ = 0 for all *k* ≠ *j*. Doing so, one can substitute this solution into (30) to yield

33 $${c}_{j}{\kappa }_{j}\exp ({\kappa }_{j}t){p}_{j}(\theta )+{c}_{j}\exp ({\kappa }_{j}t){\dot{p}}_{j}(\theta )=({J}_{{y}_{0}^{\gamma }}+\Delta {J}_{{y}_{\epsilon }^{\gamma }(t)}){c}_{j}\exp ({\kappa }_{j}t){p}_{j}(\theta )+{\mathcal{O}}({\epsilon }^{2}),$$

which simplifies to

34 $${\kappa }_{j}{p}_{j}(\theta )+{\dot{p}}_{j}(\theta )=({J}_{{y}_{0}^{\gamma }}+\Delta {J}_{{y}_{\epsilon }^{\gamma }(t)}){p}_{j}(\theta )+{\mathcal{O}}({\epsilon }^{2}).$$

Note that in the above equation, the time dependence on *θ* has been dropped for notational convenience. Asymptotically expanding *p*_*j*_(*θ*) in orders of *ϵ*, ${p}_{j}(\theta )={p}_{j}^{0}(\theta )+\epsilon {p}_{j}^{1}+{\mathcal{O}}({\epsilon }^{2})$, one can rewrite the above equation as

35 $$\left({\dot{p}}_{j}^{0}(\theta )+\epsilon {\dot{p}}_{j}^{1}(\theta )\right)=-{\kappa }_{j}({p}_{j}^{0}(\theta )+\epsilon {p}_{j}^{1}(\theta ))+({J}_{{y}_{0}^{\gamma }}+\Delta {J}_{{y}_{\epsilon }^{\gamma }})\left({p}_{j}^{0}(\theta )+\epsilon {p}_{j}^{1}(\theta )\right)+{\mathcal{O}}({\epsilon }^{2}).$$

Separating ${\mathcal{O}}(1)$ and ${\mathcal{O}}(\epsilon )$ terms in (35) yields

36 $${\dot{p}}_{j}^{0}(\theta )=({J}_{{y}_{0}^{\gamma }}-{\kappa }_{j}{\mathbb{I}}){p}_{j}^{0}(\theta )$$

37 $${\dot{p}}_{j}^{1}(\theta )=({J}_{{y}_{0}^{\gamma }}-{\kappa }_{j}{\mathbb{I}}){p}_{j}^{1}(\theta )+\frac{1}{\epsilon }\Delta {J}_{{y}_{\epsilon }^{\gamma }}{p}_{j}^{0}(\theta )$$

The functions ${p}_{j}^{0}(\theta )$ and ${p}_{j}^{1}(\theta )$ are periodic solutions of (36) and (37), respectively. Consequently, the solution to (36) is simply a constant vector in the null space of $({J}_{{y}_{0}^{\gamma }}-{\kappa }_{j}{\mathbb{I}})$. Additionally, equation (37) is a periodically forced version of the linear system from (36) since $\Delta {J}_{{y}_{\epsilon }^{\gamma }}$ is a function of *Δy*(*t*). Because neither (36) nor (37) depend on *ϵ* (recall from (31) that $\Delta {J}_{{y}_{\epsilon }^{\gamma }}$ is proportional *Δy* and hence proportional to *ϵ*), *p*_*j*_(*θ*) can be written as

38 $${p}_{j}(\theta )={p}_{j}^{0}+\epsilon (\Delta {p}_{j}(\theta )+{p}_{j}^{{\rm{shift}}})+{\mathcal{O}}({\epsilon }^{2}),$$

where ${p}_{j}^{{\rm{shift}}}\in {{\mathbb{R}}}^{n+1}$ is a constant shift that results from the fact that $({J}_{{y}_{0}}^{\gamma }-{\kappa }_{j}{\mathbb{I}})$ has a zero eigenvalue.

Next, the solution ${{\mathcal{I}}}_{j}(\theta )$ will be considered, which according to (25) has the solution

39 $$\begin{array}{lll}\frac{d{{\mathcal{I}}}_{j}(\theta )}{dt} & = & ({\kappa }_{j}{\mathbb{I}}-{J}_{{y}_{\epsilon }^{\gamma }(t)}^{T}){{\mathcal{I}}}_{j}(\theta ),\\  & = & ({\kappa }_{j}{\mathbb{I}}-{J}_{{y}_{0}^{\gamma }}^{T}-\Delta {J}_{{y}_{\epsilon }^{\gamma }(t)}^{T}){{\mathcal{I}}}_{j}(\theta )+{\mathcal{O}}({\epsilon }^{2}),\end{array}$$

where $$\Delta {J}_{{y}_{\epsilon }^{\gamma }(t)}^{T}$$ was defined in (31). As with the solution *p*_*j*_(*θ*), asymptotically expanding $${{\mathcal{I}}}_{j}(\theta )={{\mathcal{I}}}_{j}^{0}(\theta )$$$$+\epsilon {{\mathcal{I}}}_{j}^{1}(\theta )+{\mathcal{O}}({\epsilon }^{2})$$, substituting into (39) and separating the $${\mathcal{O}}(1)$$ and $${\mathcal{O}}(\epsilon )$$ terms yields

40 $${\dot{{\mathcal{I}}}}_{j}^{0}=({\kappa }_{j}{\mathbb{I}}-{J}_{{y}_{0}^{\gamma }(t)}^{T}){{\mathcal{I}}}_{j}^{0},$$

41 $${\dot{{\mathcal{I}}}}_{j}^{1}=({\kappa }_{j}{\mathbb{I}}-{J}_{{y}_{0}^{\gamma }(t)}^{T}){{\mathcal{I}}}_{j}^{1}-\frac{1}{\epsilon }\Delta {J}_{{y}_{\epsilon }^{\gamma }(t)}^{\gamma }{{\mathcal{I}}}_{j}^{0},$$

which has the same general form as (36) and (37). Additionally, the transpose does not change the eigenvalues so that $({\kappa }_{j}{\mathbb{I}}-{J}_{{y}_{0}^{\gamma }(t)}^{T})$ still has a zero eigenvalue. Therefore analogous to the *p*_*j*_(*θ*) from (38), ${{\mathcal{I}}}_{j}(\theta )$ can be written as

42 $${{\mathcal{I}}}_{j}(\theta )={{\mathcal{I}}}_{j}^{0}+\epsilon (\Delta {{\mathcal{I}}}_{j}(\theta )+{{\mathcal{I}}}_{j}^{{\rm{shift}}})+{\mathcal{O}}({\epsilon }^{2}),$$

where ${{\mathcal{I}}}_{j}^{{\rm{shift}}}\in {{\mathbb{R}}}^{n+1}$ is constant.

Finally, evaluation of (27) to obtain the reduced function ${{\mathcal{C}}}_{j}^{j}$ requires knowledge of ${H}_{i,{y}_{\epsilon }^{\gamma }(t)}$, i.e., the Hessian evaluated along the trajectory ${y}_{\epsilon }^{\gamma }(t)$. This can be found to leading order *ϵ* with the expansion

43 $${H}_{i,{y}_{\epsilon }^{\gamma }(t)}={H}_{i,{y}_{0}^{\gamma }}+\Delta {H}_{i}(\Delta y(t))+{\mathcal{O}}({\epsilon }^{2}),$$

where the *j*^th^ row and *k*^th^ column of Δ*H*_*i*_(*y*) is equal to ${\sum }_{m=1}^{n+1}\left(\frac{{\partial }^{3}{F}_{a,i}}{\partial {y}_{j}\partial {y}_{k}\partial {y}_{m}}{y}_{m}\right)$, where *F*_*a*,*i*_ is the *i*^th^ element of *F*_*a*_. Substituting this result along with (42) and (38) into (27), one finds that ${{\mathcal{C}}}_{j}^{j}(\theta )$ is the periodic solution to

44 $$\begin{array}{lll}\frac{d{{\mathcal{C}}}_{j}^{j}(\theta )}{dt} & = & -\mathop{\sum }\limits_{i=1}^{n+1}\left\{\right.\left(\right.{e}_{i}^{T}\left(\right.{{\mathcal{I}}}_{j}^{0}+\epsilon (\Delta {{\mathcal{I}}}_{j}(\theta )+{{\mathcal{I}}}_{j}^{{\rm{shift}}})\left)\right.\left)\right.\times \left(\right.{H}_{i,{y}_{0}^{\gamma }}+\Delta {H}_{i}(\Delta y(t))\left)\right.\left\}\right.\left(\right.{p}_{j}^{0}+\epsilon (\Delta {p}_{j}(\theta )+{p}_{j}^{{\rm{shift}}})\left)\right.\\  &  & -\left(\right.{J}_{{y}_{0}^{\gamma }}+\Delta {J}_{{y}_{\epsilon }^{\gamma }(t)}\left)\right.{{\mathcal{C}}}_{j}^{j}(\theta )+{\mathcal{O}}({\epsilon }^{2}).\end{array}$$

Once again, taking the asymptotic expansion ${{\mathcal{C}}}_{j}^{j}(\theta )={{\mathcal{C}}}_{j}^{j,0}(\theta )+\epsilon {{\mathcal{C}}}_{j}^{j,1}(\theta )+{\mathcal{O}}({\epsilon }^{2})$, substituting into (44) and separating ${\mathcal{O}}(1)$ and ${\mathcal{O}}(\epsilon )$ terms yields

45 $${\dot{{\mathcal{C}}}}_{j}^{j,0}(\theta )=-{\sum }_{i=1}^{n+1}\left\{\right.\left(\right.{e}_{i}^{T}{{\mathcal{I}}}_{j}^{0}\left)\right.{H}_{i,{y}_{0}}^{\gamma }{p}_{j}^{0}\left\}\right.-{J}_{{y}_{0}}^{\gamma }{{\mathcal{C}}}_{j}^{j,0}(\theta ),$$

46 $$\begin{array}{ccc}{\dot{{\mathcal{C}}}}_{j}^{j,1}(\theta ) & = & -{\sum }_{i=1}^{n+1}\{({e}_{i}^{T}\Delta {{\mathcal{I}}}_{j}(\theta )){H}_{i,{y}_{0}^{\gamma }}{p}_{j}^{0}+({e}_{i}^{T}{{\mathcal{I}}}_{j}^{0})\Delta {H}_{i}(\Delta y(t)){p}_{j}^{0}+({e}_{i}^{T}{{\mathcal{I}}}_{j}^{0}{H}_{i,{y}_{0}^{\gamma }}\Delta {p}_{j}(\theta ))\}\\  &  & -\Delta {J}_{{y}_{\epsilon }(t)}^{\gamma }{{\mathcal{C}}}_{j}^{j,0}(\theta )-{b}_{j}^{{\rm{s}}um}-{J}_{{y}_{0}}^{\gamma }{{\mathcal{C}}}_{j}^{j,1}(\theta ),\end{array}$$

where ${b}_{j}^{{\rm{s}}{\rm{u}}{\rm{m}}}={\sum }_{i=1}^{n+1}\{({e}_{i}^{T}{{\mathcal{I}}}_{j}^{{\rm{shift}}}){H}_{i,{y}_{0}^{\gamma }}{p}_{j}^{0}+({e}_{i}^{T}{{\mathcal{I}}}_{j}^{0}){H}_{i,{y}_{0}^{\gamma }}{p}_{j}^{{\rm{shift}}}\}$ is a constant. The solution to (45) is simply a constant. Equation (46) is the periodic solution to ${\dot{{\mathcal{C}}}}_{j}^{j,1}(\theta )={f}_{j}^{C}(t)-{b}_{j}^{{\rm{s}}{\rm{u}}{\rm{m}}}-{J}_{{y}_{0}}^{\gamma }{{\mathcal{C}}}_{j}^{j,1}(\theta )$ where ${f}_{j}^{C}(t)$ comprises all time dependent terms of the right hand side of (46). Much like for the previous equations, ${\dot{{\mathcal{C}}}}_{j}^{j,1}$ is the solution of a periodically forced, linear time invariant differential equation. Because of this overall structure, one can write the solution as

47 $${{\mathcal{C}}}_{j}^{j}(\theta )={{\mathcal{C}}}_{j}^{j,0}+\epsilon (\Delta {{\mathcal{C}}}_{j}^{j}(\theta )+{{\mathcal{C}}}_{j}^{j,{\rm{shift}}})+{\mathcal{O}}({\epsilon }^{2}).$$

**Figure 11:**
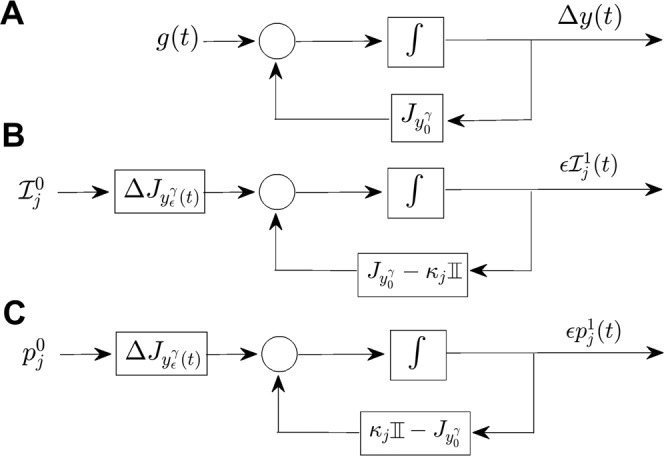
Summary of the linear relationships between the terms of the right hand side of (44) and the external forcing *g*(*t*). To leading order *ϵ*, The periodic orbit ${y}_{\epsilon }^{\gamma }(t)={y}_{0}^{\gamma }+\Delta y(t)$ is is the periodic solution to the linear system represented in panel A. The leading order *ϵ* time-varying components of the function *p*_*j*_ and ${{\mathcal{I}}}_{j}$ result from the linear relationships represented in panels B and C, respectively. Note that $\Delta {J}_{{y}_{\epsilon }}^{\gamma }(t)$ is a linear function of *Δy*(*t*). Any term that does not have explicit dependence on *t* in the above figure is constant.

Carefully examining the structure of the phase dependent term, $\Delta {C}_{j}^{j}(\theta )$, of (47) recall the initial assumption that *u*(*t*) is assumed to have the structure $u(t)=\sin (\omega t+\phi )$ with $$g(t)={\left[\begin{array}{cccc}u(t) & 0 & \ldots  & 0\end{array}\right]}^{T}$$. Figure 11 summarizes the relationships between *g*(*t*) and the order *ϵ* accurate time varying inputs to the right hand side of (44) from equations (28), (37), and (41). The term Δ*y*(*t*) is the periodic solution to the relationship represented in panel A. The term $\Delta {J}_{{y}_{\epsilon }}^{\gamma }(t)$ is a linear function of Δ*y*(*t*), so that $\Delta {{\mathcal{I}}}_{j}(\theta (t))$ and Δ*p*_*j*_(*θ*(*t*)) are periodic solutions resulting from the linear relationships represented in panels B and C. Furthermore, Δ*H*_*i*_ is a linear function of Δ(*y*). Taken together, all of the time varying inputs to the right hand side of (46) must be sinusoids with period 2*π*/*ω*. Consequently, because (44) is linear, This implies that the non-constant terms of the solution ${{\mathcal{C}}}_{j}^{j}(\theta )$, i.e. $\Delta {{\mathcal{C}}}_{j}^{j}$ can be written as

48 $$\Delta {{\mathcal{C}}}_{j}^{j}(\theta (t))={\chi }_{j}(T)\sin (\omega t+\phi +{\beta }_{j}(T)),$$

where *χ*_*j*_(*T*) and *β*_*j*_(*T*) are vectors that characterize the gain and phase shift due to the linear relationships that are used to determine ${{\mathcal{C}}}_{j}^{j}(\theta (t))$ from the input *u*(*t*). Recall that this result is valid for a purely sinusoidal input $u(t)=\sin (\omega t+\phi )$, however, due to system linearity more complicated inputs can be considered relatively easily. To do so consider the Fourier expansion of a general *T*-periodic, continuous, and piecewise smooth input $u(t)={\sum }_{k=1}^{\infty }\sqrt{{a}_{k}^{2}+{b}_{k}^{2}}\sin (\omega kt+{\phi }_{k})$, where *a*_*k*_ and *b*_*k*_ are Fourier coefficients and *ϕ*_*k*_ is determined by the relative contribution of each coefficient. Due to linearity of the relationships used to determine $\Delta {{\mathcal{C}}}_{j}^{j}(\theta (t))$ in (47), one can extend the relation from (48) to write

49 $$\Delta {{\mathcal{C}}}_{j}^{j}(\theta (t))={\sum }_{k=1}^{\infty }\sqrt{{a}_{k}^{2}+{b}_{k}^{2}}\left[{\chi }_{j}(T/k)\sin (\omega kt+{\phi }_{k}+{\beta }_{j}(T/k))\right].$$

### Change in floquet exponents in response to a periodic stimulus

After the derivation of (47) and (49), one can use (23) from^78^ to characterize the change in Floquet exponents. Specifically, consider a nominal input *qu*(*t*), and an infinitesimally shifted input (*q* + *dq*)*u*(*t*). According to (23), the resulting leading order *ϵ*^2^ accurate infinitesimal change to the Floquet exponent due to the infinitesimal perturbation *dq* is

50 $$\begin{array}{lll}d{\kappa }_{j} & = & \frac{dq}{T}{\int }_{0}^{T}{C}_{j}^{j}(\omega t)u(t)dT,\\  & = & \frac{dq}{T}{\int }_{0}^{T}\left(\right.{C}_{j}^{j,{\rm{const}}}+\Delta {C}_{j}^{j}(\omega t)\left)\right.u(t)dT,\\  & = & \frac{dq}{T}{\int }_{0}^{T}\Delta {C}_{j}^{j}(\omega t)u(t)dT.\end{array}$$

in the second line above, ${C}_{j}^{j,{\rm{const}}}$ (resp., $\Delta {C}_{j}^{j}$) represents the sum of all of the constant (resp., periodic) terms from ${e}_{1}^{T}{{\mathcal{C}}}_{j}^{j}(\omega t)$ as calculated from (47). In the third line, these constant terms disappear when integrated over one period of the zero-mean periodic stimulus *u*(*t*). Further simplifying (50) using (49) and substituting the Fourier expansion of *u*(*t*) yields

51 $$\begin{array}{ccc}d{\kappa }_{j} & = & \frac{dq}{T}{\int }_{0}^{T}{\sum }_{k=1}^{{\rm{\infty }}}(\sqrt{{a}_{k}^{2}+{b}_{k}^{2}}[{e}_{1}^{T}{\chi }_{j}(T/k)\sin (\omega kt+{\phi }_{k}+{e}_{1}^{T}{\beta }_{j}(T/k))])\\  &  & {\times \sum }_{j=1}^{{\rm{\infty }}}(\sqrt{{a}_{j}^{2}+{b}_{j}^{2}}\sin (\omega jt+{\phi }_{j}))dt,\\  & = & \frac{dq}{T}{\int }_{0}^{T}{\sum }_{k=1}^{{\rm{\infty }}}{\sum }_{j=1}^{{\rm{\infty }}}(\sqrt{{a}_{k}^{2}+{b}_{k}^{2}}[{e}_{1}^{T}{\chi }_{j}(T/k)\sin (\omega kt+{\phi }_{k}+{e}_{1}^{T}{\beta }_{j}(T/k))]\\  &  & \sqrt{{a}_{j}^{2}+{b}_{j}^{2}}\sin (\omega jt+{\phi }_{j}))dt.\end{array}$$

Recall that ${C}_{j}^{j}(\omega t)$ is the first element of ${{\mathcal{C}}}_{j}^{j}$ so that ${e}_{1}^{T}$ is used in the above equation to obtain the first elements of *β*_*j*_ and *χ*_*j*_. In order to further evaluate (51) it will be necessary to bring the integral inside of the infinite sums. This can be done if the sums converge absolutely. To show that this is indeed the case here, first note that Δ*y* (and consequently *χ*_*j*_ asymptotically approaches zero as *T* tends to zero. Then, let *M* be an upper bound for ${e}_{1}^{T}{\chi }_{j}(T/k)$ for *k* = 1, …, *∞*. We can then write

52 $$\begin{array}{ccc}d{\kappa }_{j} & = & \frac{dq}{T}{\int }_{0}^{T}{\sum }_{k=1}^{{\rm{\infty }}}{\sum }_{j=1}^{{\rm{\infty }}}(\sqrt{{a}_{k}^{2}+{b}_{k}^{2}}[{e}_{1}^{T}{\chi }_{j}(T/k)\sin (\omega kt+{\phi }_{k}+{e}_{1}^{T}{\beta }_{j}(T/k))]\\  &  & \sqrt{{a}_{j}^{2}+{b}_{j}^{2}}\sin (\omega jt+{\phi }_{j}))dt,\\  & \le  & \frac{Mdq}{T}{\int }_{0}^{T}{\sum }_{k=1}^{{\rm{\infty }}}{\sum }_{j=1}^{{\rm{\infty }}}(\sqrt{{a}_{k}^{2}+{b}_{k}^{2}}\sqrt{{a}_{j}^{2}+{b}_{j}^{2}})dt,\\  & = & \frac{Mdq}{T}{\int }_{0}^{T}{\sum }_{k=1}^{{\rm{\infty }}}(\sqrt{{a}_{k}^{2}+{b}_{k}^{2}}){\sum }_{j=1}^{{\rm{\infty }}}(\sqrt{{a}_{j}^{2}+{b}_{j}^{2}})dt.\end{array}$$

The infinite sums comprising Fourier series coefficients in (52) converge and hence, (51) absolutely convergent. This allows one to bring the integral inside the infinite sums from (51). Further manipulation yields

53 $$\begin{array}{ccc}d{\kappa }_{j} & = & \frac{dq}{T}\mathop{\sum }\limits_{k=1}^{{\rm{\infty }}}\mathop{\sum }\limits_{j=1}^{{\rm{\infty }}}{\int }_{0}^{T}(\sqrt{{a}_{k}^{2}+{b}_{k}^{2}}[{e}_{1}^{T}{\chi }_{j}(T/k)\sin (\omega kt+{\phi }_{k}+{e}_{1}^{T}{\beta }_{j}(T/k))]\sqrt{{a}_{j}^{2}+{b}_{j}^{2}}\\  &  & \sin (\omega jt+{\phi }_{j}))dt,\\  & = & \frac{dq}{T}\mathop{\sum }\limits_{k=1}^{{\rm{\infty }}}{\sum }_{j=1}^{{\rm{\infty }}}({e}_{1}^{T}{\chi }_{j}(T/k)\sqrt{{a}_{k}^{2}+{b}_{k}^{2}}\sqrt{{a}_{j}^{2}+{b}_{j}^{2}}\\  &  & {\int }_{0}^{T}[\sin (\omega kt+{\phi }_{k}+{e}_{1}^{T}{\beta }_{j}(T/k))]\sin (\omega jt+{\phi }_{j}))dt,\\  & = & 2dq\mathop{\sum }\limits_{k=1}^{{\rm{\infty }}}({a}_{k}^{2}+{b}_{k}^{2}){\alpha }_{i}(T/k),\end{array}$$

where

54 $${\alpha }_{i}(y)\equiv \frac{{e}_{1}^{T}\chi (y)}{2y}{\int }_{0}^{y}\sin (2\pi t/y+{e}_{1}^{T}{\beta }_{j}(y))\sin (2\pi t/y)dt.$$

Finally, using the relationship from (53), one can show that the overall change resulting from the input *ϵu*(*t*) is

55 $$\begin{array}{lll}\Delta {\kappa }_{j} & = & {\int }_{0}^{\epsilon }2q\mathop{\sum }\limits_{k=1}^{\infty }({a}_{k}^{2}+{b}_{k}^{2}){\alpha }_{i}(T/k)dq\\  & = & {\epsilon }^{2}\mathop{\sum }\limits_{k=1}^{\infty }({a}_{k}^{2}+{b}_{k}^{2}){\alpha }_{i}(T/k)\end{array}$$

The relationship (55) can be written in the same form as (3) from the results section by recalling that *κ*_*j*_ = *λ*_*j*_ when *u* = 0 so that Δ*κ*_*j*_ = *κ*_*j*_ − *λ*_*j*_. Additionally, in (3), the *ϵ*^2^ from (55) is pulled into the Fourier coefficients.

### Simplified model of the shannon-puglisi-bers model during conduction block

During conduction block induced by periodic stimulation of the Shannon-Puglisi-Bers model^54^ the ionic buffering variables and ionic concentrations are nearly static. In order to aid in the analysis, it is assumed that these variables remain constant during conduction block. This simplification results in a 14-variable model comprised of gating variables *m*, *h*, *j*, *d*, *f*, *X*_to_, s, *X*_to_, f, *Y*_to_, s, *Y*_to_, f, *X*_r_, *X*_s_, two copies of the gating variable *f*_CaB_ (one for each of the sarcolemmal and junctional spaces), and the transmembrane voltage *V*. The remaining variables are taken to be constants found according to the following procedure: Step 1) Pace the full model (13) with a period of one second until steady state behavior is reached. Step 2) After the next action potential is initiated, apply the periodic stimulation ${I}_{{\rm{stim}}}(t)=10\sin (2\pi t/10)$ for 2 seconds of simulation time. This magnitude of stimulation is large enough to ensure that repolarization block occurs in the model. Step 3) Values which are assumed to be constant in the preliminary reduction are found by averaging over the final 1 second of simulation from Step 2.
